# Supplementary material for: A Self-Management Programme of Activity Coping and Education - SPACE for COPD(C) - in primary care: The protocol for a pragmatic trial
Source: BMJ Open. 2017 Jul 10;7(7):e014463. doi: 10.1136/bmjopen-2016-014463 (PMC5734249; doi:10.1136/bmjopen-2016-014463)
Supplement: Author's manuscript [file bmjopen-2016-014463.draft_revisions.pdf]

BMJ Open

A self-management programme of activity coping and education - SPACE FOR COPD© - in primary care: The protocol for a pragmatic trial

Journal:	*BMJ Open*
Manuscript ID	bmjopen-2016-014463
Article Type:	Protocol
Date Submitted by the Author:	26-Sep-2016
Complete List of Authors:	Bourne, Claire; University Hospitals of Leicester NHS Trust, Centre of Exercise and Rehabilitation Science<br>Kanabar, Pratiksha; University Hospitals of Leicester NHS Trust, Centre of Exercise and Rehabilitation Science, Respiratory Biomedical Research Unit, Glenfield Hospital<br>Mitchell, Katy; University Hospitals of Leicester NHS Trust, Centre of Exercise and Rehabilitation Science, Respiratory Biomedical Research Unit, Glenfield Hospital<br>Schreder, Sally; University Hospitals of Leicester NHS Trust, Centre of Exercise and Rehabilitation Science; University of Leicester Diabetes Research Centre, Air Zone<br>Houchen-Wolloff, Linzy; University Hospitals of Leicester NHS Trust, Centre of Exercise and Rehabilitation Science, Respiratory Biomedical Research Unit, Glenfield Hospital<br>Bankart, M. John; Keele University, Institute of Primary Care<br>Apps, Lindsay ; Leicester Respiratory Biomedical Research Unit, Centre for Exercise and Rehabilitation Science, Respiratory Biomedical Research Unit, Glenfield Hospital<br>Hewitt, Stacey; University Hospitals of Leicester NHS Trust, Centre of Exercise and Rehabilitation Science, Leicester Respiratory Biomedical Research Unit, Respiratory Biomedical Research Unit, Glenfield Hospital<br>Harvey-Dunstan, Theresa; University Hospitals of Leicester NHS Trust, Centre of Exercise and Rehabilitation Science, Leicester Respiratory Biomedical Research Unit, Respiratory Biomedical Research Unit, Glenfield Hospital<br>Singh, Sally; University Hospitals of Leicester NHS Trust, Cardiac/Pulmonary Rehabilitation; Loughborough University, National Centre for Sport and Exercise Medicine
**Primary Subject Heading**:	Respiratory medicine
Secondary Subject Heading:	Patient-centred medicine
Keywords:	Chronic Obstructive Pulmonary Disease, Self-Management Support, PRIMARY CARE

For peer review only

A self-management programme of activity coping and education - SPACE FOR COPD© - in primary care: The protocol for a pragmatic trial

C. Bourne[1],P. Kanabar[1], K. Mitchell[1], S. Schreder[1], L. Houchen-Wolloff[1], J. Bankart[2], L. Apps[1], S. Hewitt[1], T. Harvey-Dunstan[1], S. Singh[1, 3]

[1]Collaboration and Leadership for Applied Health Research and Care - East Midlands, Centre of Exercise and Rehabilitation Science, Respiratory Biomedical Research Unit, Glenfield hospital, Groby Road, Leicester, Leicestershire, UK, LE3 9QP

[2]Department of Primary Care and Health Sciences, Keele University, Keele, UK

[3]National Centre for Sport and Exercise Medicine, Loughborough University, Leicestershire, UK, LE11 3TU

Corresponding Author:

Dr Claire Bourne

Centre of Exercise and Rehabilitation Science,

Respiratory Biomedical Research Unit,

Glenfield hospital,

Groby Road,

LE3 9QP

Email: Claire.Bourne@uhl-tr.nhs.uk

Tel: 0116 258 3035

Key words: COPD, self-management support, primary care

Word count: 3,906 words

For peer review only

For peer review only - http://bmjopen.bmj.com/site/about/guidelines.xhtml

ABSTRACT

Introduction

National guidance for COPD suggests that self-management support be provided for patients. Our institution has developed a standardised, manual-based, supported self-management programme: Self-Management Programme of Activity Coping and Education - SPACE for COPD©. SPACE was previously piloted on a 1-2-1 basis, delivered by researchers, to individuals with COPD. Discussions with stakeholders highlighted considerable interest in delivering the SPACE FOR COPD© intervention as a group-based self-management programme facilitated by healthcare professionals (HCPs) in primary care settings. The study aims are to explore the feasibility, acceptability and efficacy for the intervention to be delivered and supported by HCP's rather than researchers and experienced clinicians; and to examine whether group-based delivery of SPACE FOR COPD©, with sustained support, improves the maintenance of outcomes following the SPACE FOR COPD© intervention.

Methods and Analysis

A prospective, multi-site, single-blinded Randomised Controlled Trial (RCT) will be conducted, with follow-up at 6 and 9 months. Participants will be randomly assigned to either the control group (usual care) or intervention group (a 6-session, group-based SPACE for COPD© self-management programme delivered over 5 months). The primary outcome is change in COPD Assessment Test at 6-months.

For peer review only - http://bmjopen.bmj.com/site/about/guidelines.xhtml

A discussion session will be conducted with HCP’s who deliver the intervention to discuss and gain insight into any potential facilitators/barriers to implementing the intervention in practice. Furthermore, we will conduct semi-structured focus groups with intervention participants to understand feasibility and acceptability. All qualitative data will be analysed thematically.

Ethics and Dissemination

The project has received a favourable opinion from South Hampshire B Research Ethics Committee, REC reference: 14/SC/11/69 and full R&D approval from the University Hospitals of Leicester NHS Trust: 152408.

Study results will be disseminated through appropriate peer reviewed journals, national and international respiratory/physiotherapy conferences, via the Collaboration and Leadership in Applied Health Research and Care and through social media.

Trial registration: ISRCTN17942821.

Protocol Version: 11 18.11.2015

Key words: COPD, self-management support, primary care

Strengths and Limitations:

- The study concentrates on the feasibility, acceptability and efficacy of a group-based self-management support intervention (Self-Management

For peer review only

For peer review only - http://bmjopen.bmj.com/site/about/guidelines.xhtml

Programme of Activity Coping and Education - SPACE for COPD©), delivered and supported by healthcare professionals in community settings.

- Much of the success depends on the willingness and ability of the participants to take part in, and travel to, group-based self-management support sessions in the community.
- In order to understand how the intervention could be delivered within existing health services and identify key barriers and facilitators to its implementation, the study will compare a variety of clinical and non-clinical, quantitative and qualitative outcomes between a group-based self-management support intervention and usual care.

For peer review only

For peer review only - http://bmjopen.bmj.com/site/about/guidelines.xhtml

INTRODUCTION

COPD is the third leading cause of death worldwide, and is associated with considerable disability, impaired quality of life and high utilisation of healthcare resources (1). Symptoms and manifestations of the disease can be modified by adopting appropriate health behaviours, including but not limited to, exercise, physical activity, smoking cessation, anxiety management, breathing control, medication adherence and exacerbation management (2). Acknowledging the importance of the role of the patient in adopting these behaviours, there has been a shift in attitude from a traditional paternalistic model of care towards a more collaborative approach for chronic disease management. The NHS Five Year Forward View's aim is for the NHS to become better at helping people to manage their own health by staying healthy, making informed choices of treatment, managing conditions and avoiding complications (3). Inevitably, the patient is predominantly responsible for administering their own care, and making choices about health behaviours which will affect their outcomes. Self-management support aims to inform and support patients in making these choices. National and international guidelines for the management of COPD suggest that self-management support should be provided for people with COPD, though at present evidence for how and when that support should be delivered is less robust (2).

Reports in the literature describe programmes that have targeted interventions for patients who have been hospitalised with a COPD-related admission, often with the primary ambition of reducing future admissions (4). These studies have had little impact on readmission. Arguably, the offer of supported self-management should be offered earlier in the disease trajectory. Other COPD self-management programmes

For peer review only - http://bmjopen.bmj.com/site/about/guidelines.xhtml

beyond the UK have been described in a stable population. Although the models of care delivered are quite heterogeneous (5), with some programmes providing up to two years of weekly supervised exercise training and education (3, 6-8). The infrastructure and resources required to provide such comprehensive support means they are unlikely to be deliverable to the breadth of the COPD population in the UK. In order to address this, we previously developed and tested a new self-management programme which offered a 'light touch' approach so that it could be provided on a larger scale.

A Self-management Programme of Activity Coping and Education – SPACE FOR COPD© – aims to support people with COPD in managing day-to-day tasks, minimise symptom burden, provoke health enhancing behaviour change and enhance emotional well-being. The programme is structured around the SPACE FOR COPD© manual, which combines both generic self-management skills and disease-specific tasks. Pilot testing assessed the feasibility and acceptability of the intervention to patients (9), and a fully powered randomised controlled trial assessed the efficacy of the intervention in primary care (10), powered for change in symptom burden measured by the self-reported Chronic Respiratory Questionnaire (CRQ-SR) dyspnoea domain at six months. In these studies the SPACE FOR COPD© manual was introduced to patients during an initial consultation with a healthcare professional (using motivational interviewing techniques), followed by two telephone calls during the subsequent six weeks. Secondary outcomes included other domains of the CRQ-SR, shuttle walking tests, disease knowledge, anxiety, depression, self-efficacy, smoking status and healthcare utilisation measured at baseline, six weeks and six months follow-up. Results demonstrated significant short-term improvements

For peer review only - http://bmjopen.bmj.com/site/about/guidelines.xhtml

in CRQ-SR dyspnoea, anxiety, fatigue and emotion scores, exercise performance, and disease knowledge. At six months, anxiety, exercise performance and smoking status outcomes remained significantly different between the intervention group and the usual care group, though there was no between-group difference in change in CRQ-SR dyspnoea.

Implementation focussed work carried out following these studies with healthcare professionals in primary care and local Clinical Commissioning Groups (CCGs), demonstrated considerable interest in delivering the SPACE FOR COPD© intervention as a group-based intervention rather than on a one-to-one basis. The most common theoretical rationale underpinning delivery of group-based self-management support interventions is Social Cognitive Theory/Social Learning Theory (11, 12). Bandura's (1977, 1997) social learning theory posits that behaviour is influenced by beliefs about one's ability to perform a particular behaviour (self-efficacy expectations), beliefs about the effectiveness of the behaviour (e.g., the advantages and disadvantages of performing this behaviour; outcome expectations) and learning through social observation (including social norms, social support or pressure, and the behaviours of others). Peer support and use of other patients as role models are approaches grounded in this theory, and directly applicable to group-based self-management support interventions.

Delivery of SPACE for COPD© as a group-based intervention allows for several face-to-face contacts between patients and healthcare professionals over a number of sessions. These contacts could be spread out further across a longer period, which may be more successful in maintaining behaviour change. Furthermore,

For peer review only - http://bmjopen.bmj.com/site/about/guidelines.xhtml

having earlier sessions closer together in time allows group cohesion to take place, an important factor in optimising group dynamics (13).

The SPACE for COPD© intervention has also previously been delivered by a member of the research team rather than by existing clinical services. If the group-based intervention were to be implemented in primary care following the current study, importance would be placed on delivery by healthcare professionals in a format that is feasible and acceptable to healthcare professionals, health service providers and to patients. Understanding how this intervention can be delivered within existing health services and identifying key barriers and facilitators to its implementation is an important next step in the development of this complex intervention.

Objectives of the study

1. To examine whether group-based delivery of SPACE FOR COPD©, with sustained support, improves the maintenance of outcomes following the intervention.
2. To explore feasibility, acceptability and efficacy of the intervention to be delivered and supported by HCP's rather than researchers.
3. To explore HCP's experiences of delivering the intervention and identify any barriers to delivery in practice.
4. To understand, from the patient perspective, the feasibility and acceptability of the SPACE for COPD© intervention delivered by HCPs in a group-based, community setting.

For peer review only - http://bmjopen.bmj.com/site/about/guidelines.xhtml

METHODS/DESIGN

Study design

The trial is a prospective, multi-site, single (assessor) blinded randomised controlled trial comparing a community based, HCP led, group-based self-management programme based on the SPACE for COPD© manual with usual care. The design of the study and flow of participants is described in figure 1. The study will be run across Leicestershire and Rutland, and a total of 150 participants will be recruited (75 in the intervention group and 75 in the control group).

Recruitment of participants

We will recruit participants with COPD, who will be identified from primary care (GP) COPD registers and from patients who respond to a poster advertisement that will be displayed at GP practices and hospitals. We will also recruit participants from the following areas within the Respiratory Biomedical Research Unit at University Hospitals of Leicester:

- Those who have been involved in previous research trials who have agreed to be contacted again; or
- Those who were unsuitable for previous research trials, but who agreed to be contacted about future research trials for which they might be eligible.

Participant invitation

Eligible individuals identified as having an established diagnosis of COPD are sent an invitation letter, a patient information sheet about the study, and a reply slip. For those recruited directly from primary care, the invitation letters are sent by the primary care practice where the search was conducted. For those recruited from

For peer review only - http://bmjopen.bmj.com/site/about/guidelines.xhtml

existing databases, the invitation is sent from the Principal Investigator of the study. Individuals who are interested in taking part are asked to return a reply slip directly to the SPACE for COPD© research team. Interested participants are then contacted via their preferred contact method and an appointment is arranged for a baseline visit.

Eligibility criteria

Participants are eligible for the trial if they have:

- An established diagnosis of Chronic Obstructive Pulmonary Disease (COPD) as defined by The Global Initiative for Chronic Obstructive Lung Disease (GOLD) criteria.

Patients are excluded from participating in the trial if they are:

- Unable to participate in the exercise component of the SPACE for COPD© programme due to neurological, locomotive, or psychiatric disability; or other comorbidities.
- Unable to read/write English to the level of an eight year old
- Unwilling to be randomised
- Previous participants of Pulmonary Rehabilitation or have received the SPACE for COPD© manual in the previous 12 months.

Randomisation

Once participants have consented to take part in the study and spirometry has confirmed a COPD diagnosis, participants are randomised by an un-blinded member of the study team using an online randomisation tool (sealed envelope;(14). Individuals are randomised (1:1) to the control group or the intervention (SPACE

For peer review only - http://bmjopen.bmj.com/site/about/guidelines.xhtml

FOR COPD© group-based self-management programme) group. The system randomises patients in random permuted blocks. This allows for the 1:1 ratio, but due to the random permuted blocks of 2, 4, or 6 ensures full randomisation. Simple randomisation has been chosen as there has been no requirement to stratify by age, gender, location, or other variables. Participants are immediately informed of their allocated treatment by an un-blinded member of the study team. Un-blinding is permissible in the case of medical emergencies (e.g., cardiac arrest) or patients being admitted to hospital for an exacerbation.

Study Interventions

Usual Care (Control Group)

Participants in the control group will continue with any usual check-ups/reviews and there will be no additional care provided or removed from their current access. If patients are referred to pulmonary rehabilitation in the duration of their time in the study, they will not be denied access to the programme; however, they will not be included in the final analysis due to the use of 'intention-to-treat' analysis. No additional advice, information or recommendations will be provided to participants in this group.

SPACE for COPD© group-based self-management programme

Participants in the intervention group receive a SPACE for COPD© manual and asked to attend the SPACE for COPD© group-based self-management programme usually within one month of their baseline appointment. The aim of the SPACE for

For peer review only - http://bmjopen.bmj.com/site/about/guidelines.xhtml

COPD© programme is to support people with COPD in managing day-to-day tasks, minimise symptom burden, provoke health enhancing behaviour change and enhance emotional well-being. The programme is structured around the SPACE FOR COPD© manual, which combines both generic self-management skills and disease-specific tasks. The programme is facilitated by two trained healthcare professionals (e.g., physiotherapists, respiratory specialist nurses, occupational therapists, health psychologists) to groups of up 10 participants, and delivered through six, two hour sessions, over a five month period. These sessions will be held at community venues, at times and locations to suit participants of the group to increase retention. Participants are liaised with in regards to preferences on timings and location of the group sessions to increase retention, and engagement in the intervention. The content of the programme and accompanying self-management components (15), are presented in Tables 1 and 2.

Participants will also be asked to complete the exercise component of the manual at home in their own time. A full description of the rationale, development and efficacy of the work underpinning the SPACE for COPD© manual is detailed elsewhere (9).

For peer review only

For peer review only - http://bmjopen.bmj.com/site/about/guidelines.xhtml

Table 1: SPACE for COPD© self-management programme session outline

**Session 1 (Week 1)**	**Introduction to SPACE FOR COPD©**
1	Welcome and introductions
2	Group responsibilities
3	What does it mean to have COPD?
4	What is self-management?
5	How to use the SPACE for COPD manual
6	Goal setting
7	Home activities for next session
8	Summary and close
**Session 2 (Week 2)**	**Introducing exercise and managing shortness of breath**
1	Welcome back
2	Solution focused goal feedback
3	Managing shortness of breath
4	Introduction to the walking programme
5	Goal setting
6	Activities for next session
7	Summary and close
**Session 3 (Week 4)**	**Continuing Exercise and Saving Energy**
1	Welcome back
2	Solution focused goal feedback
3	Saving your energy
4	Strength training
5	Goal setting
6	Home activities for next session
7	Group discussion
8	Summary and close
**Session 4 (Week 8)**	**Managing Stress and Emotions and the COPD Action Plan**
1	Welcome back
2	Solution focused goal feedback
3	Managing stress and emotions
4	Action plans
5	Goal setting
6	Activities for next session
7	Summary and close
**Session 5 (Week 14)**	**Question and Answer**
1	Welcome back
2	Solution focused goal feedback
3	Question and Answer
4	Goal setting
5	Activities for next session
6	Summary and close
**Session 6 (Week 20)**	**Keeping going from here**

For peer review only - http://bmjopen.bmj.com/site/about/guidelines.xhtml

1	Welcome back
2	Solution focused goal feedback
3	Hobbies
4	Maintaining exercise
5	Sharing success
6	Summary and close

For peer review only

For peer review only - http://bmjopen.bmj.com/site/about/guidelines.xhtml

Table 2: Taxonomy components present in the SPACE for COPD© facilitator manual, elaboration of the techniques under each component, direct examples from the SPACE for COPD© facilitator manual and the dose of the component

Taxonomy component	Elaboration	Examples from the SPACE for COPD facilitator manual (*dose*)
**A2. Information about available resources**		Participants are provided with information throughout the programme (*every week*).
**A3. Provision of/agreement on specific clinical action plans and/or rescue medication**		*Session 4:* Action plans (*session 4 only*).
**A6. Practical support with adherence (medication or behavioural)**	Walking and strength training diaries are provided for participants and discussed during sessions.	Walking and strength training diaries are provided for participants and discussed during solution focused goal feedback at the beginning of sessions 3-6 (*sessions 3-6*).
**A8. Safety netting**	Participants are able to call programme facilitators between sessions if needed	Participants are provided with contact details for programme facilitators who they can call if needed (*this is a constant throughout the programme*).
**A11. Training/rehearsal for practical self-management activities**	Including:<br>• managing shortness of breath<br>• saving your energy	*Session 2:* Managing shortness of breath (*session 2 only*).<br>*Session 3:* Saving your energy (*session 3 only*).
**A12. Training/rehearsal in psychological strategies**	Including:<br>• goal setting (including action planning)<br>• solution focussed goal feedback<br>• problem solving<br>• self-reward and social reward<br>• managing stress and emotions	Goal setting activity (including action planning) and solution focussed goal feedback (*once every week*).<br><br>Problem solving (*this is a constant throughout the programme*).<br><br>*Session 4:* Managing stress and emotions (*session 4 only*).
**A13. Social support**	Including:<br>• practical support<br>• emotional support	Participants are encouraged to share experiences, advice, ideas and support each other (*this is a constant throughout the programme*).

For peer review only - http://bmjopen.bmj.com/site/about/guidelines.xhtml

**A14. Lifestyle advice and support**	Including:<br>• introduction to the walking programme<br>• strength training<br>• hobbies<br>• maintaining exercise	*Session 2:* Introduction to the walking programme (*session 2 only*).<br><br>*Session 3:* Strength training (*session 3 only*).<br>*Session 6:* Hobbies (*session 6 only*).<br>*Session 6:* Maintaining exercise (*session 6 only*).

For peer review only

For peer review only - http://bmjopen.bmj.com/site/about/guidelines.xhtml

Intervention fidelity

Intervention facilitators are registered health professionals (physiotherapists, respiratory nurse specialists, health psychologists). In total, 8 registered health professionals were trained to deliver the SPACE for COPD© group-based intervention by two Health Psychologists.

All facilitators attended a one day training course to ensure that they understood the theories and philosophy underpinning the SPACE for COPD© group-based programme and the content and resources used within it. All facilitators were given a facilitator manual to support their delivery of the programme and given the opportunity to practise delivering at least one activity from the manual during the training session.

Quality Assurance

Quality assurance will be undertaken to assess delivery of intervention content and educational style. Intervention fidelity checklists for intervention facilitators and trained observers have been specifically designed for the study. Intervention facilitators will complete checklists at the end of each self-management group session, and one of the trainers will observe one session per self-management group, completing their own checklist. Intervention facilitators will receive written and verbal feedback from the trained assessor.

For peer review only - http://bmjopen.bmj.com/site/about/guidelines.xhtml

Study Outcomes

Data is collected during baseline, 6-month and 9-month appointments at the Leicester Respiratory Biomedical Research Unit by trained members of the study team. Data are collected following standardised operating procedures. Written informed consent is obtained from all participants prior to the commencement of data collection. Details of all clinical assessments and outcome measures are provided in table 3. General practitioners are informed of patients' participation in the trial and any relevant results. Any serious adverse events will be reported to the sponsor and patients' ability to exercise safely will be monitored.

For peer review only

For peer review only - http://bmjopen.bmj.com/site/about/guidelines.xhtml

Table 3: Details of study clinical assessments and outcome measures at all appointments

Baseline appointment (blinded and un-blinded study team members)	6-month appointment (blinded study team member)	9-month appointment (blinded or un-blinded study team member)
Consent<br>Collection of demographic details and medical history<br>Blood Pressure<br>Spirometry<br>Randomisation*<br>Questionnaires (CAT, EQ-5D, CRQ-SR, BCKQ, PAM, HADS)*<br>Shuttle walking tests* (intervention participants only): 2xISWT; 1xESWT<br>Participants given Senswear activity monitor to wear for 7 days*	Check consent<br>Questionnaires (CAT, EQ-5D, CRQ-SR, BCKQ, PAM, HADS)<br>Shuttle walking tests†<br>Participants given Senswear activity monitor to wear for 7 days	Check consent<br>Questionnaires (CAT, EQ-5D, CRQ-SR, BCKQ, PAM, HADS)<br>Shuttle walking tests<br>Participants given Senswear activity monitor to wear for 7 days

Key:
CAT: COPD Assessment Test; EQ-5D: European Quality of Life-5 Dimensions; CRQ-SR: Chronic Respiratory Questionnaire; BCKQ: Bristol COPD Knowledge Questionnaire; PAM: Patient Activation Measure; HADS: Hospital Anxiety and Depression Questionnaire.
ISWT: Incremental Shuttle Walking Test; ESWT: Endurance Shuttle Walking Test
*Carried out by an un-blinded member of the study team
†ESWT carried out by an un-blinded member of the study team

For peer review only

Primary Outcome

The primary outcome is health status, as measured by the COPD Assessment Test (CAT;(16) at 6-months post baseline. The CAT is a validated, short (8-item) and simple patient completed questionnaire, and assesses globally the impact of COPD (cough, sputum, dyspnea, chest tightness) on health status. The CAT is scored 0-5 with a range of 0-40; scores of 0-10, 11-20, 21-30, 31-40 represent mild, moderate, severe or very severe clinical impact.

Secondary Outcomes

Clinical measures

Exercise Capacity

Maximal exercise capacity will be measured with the incremental shuttle walk test (ISWT) according to the protocol of Singh et al.,(17) using a 10-m course. According to current standard, an individual change of at least 47.5 m is considered clinically important (18). Endurance capacity was measured with the endurance shuttle walk test (ESWT) using a 10-m course and a walking speed of 85% of the maximal ISWT walking speed (19).

Physical Activity

Physical activity is assessed using physical activity monitors. The 'Bodymedia Sensewear' (APC Cardiovascular, UK) activity monitor is a biaxial accelerometer that can report a number of parameters including step count and energy expenditure. We will also use this data to assess compliance to the physical activity recommendation

For peer review only - http://bmjopen.bmj.com/site/about/guidelines.xhtml
For peer review only

of undertaking at least 150 minutes of moderate intensity physical activity per week in bouts of at least 10 minutes. Participants are asked to wear the activity monitor on the back of their right arm for seven consecutive days (24 hours a day if possible) following their baseline, 6-month and 9-month visits.

Questionnaires

Health related quality of life

Health related quality of life data will be measured using the European Quality of Life-5 Dimensions (EQ-5D;(20,21)). The EQ-5D is a standardized questionnaire that was developed for use as a measure of health outcomes and defines health in terms of five dimensions: mobility, self-care, usual activities, pain or discomfort, and anxiety or depression.

Chronic Respiratory Questionnaire (CRQ-SR)

Disease specific health related quality of life (HRQoL) will be measured by the self-administered standardised CRQ-SR (22). An individual change of at least 0.5/domain (dyspnoea, fatigue, emotional functioning, mastery) is considered clinically important (23). There is both an initial and follow-up version depending on time of administration.

Anxiety and Depression

Depression and anxiety will be measured using the Hospital Anxiety and Depression (HADS) Scale, to produce independent subscales for anxiety and depression (24).

For peer review only - http://bmjopen.bmj.com/site/about/guidelines.xhtml

The HADS is a self-report rating scale of 14 items on a four-point Likert scale range (0–3). The HADS is a validated and a widely used questionnaire for screening for the separate dimensions of anxiety and depression and possible occurrence of anxiety and depression from patients (25) and the general population (26). It measures anxiety and depression (seven items for each subscale). Cronbach's coefficient was 0.884, which indicates good reliability. Published cut-off scores for clinically relevant indications of depression and anxiety recommend a score of 8 for each subscale (27).

Patient Activation

Patient activation (participants' knowledge, skill, and confidence for managing their own health and health care) will be measured using the Patient Activation Measure (PAM;(28). This is a 13-item patient-reported measure that has been validated in the United Kingdom as powerful and reliable measure of patient activation. Participants indicate their level of agreement on a four-point scale (strongly disagree to strongly agree) and responses are added to yield a raw score between 13 and 52. The raw score is calibrated to an activation score between 0 and 100 (the higher the score the higher the level of activation), which is then used to classify participants into one of four levels of activation (level 1: low activation; level 4: high activation).

COPD Knowledge

The Bristol COPD Knowledge Questionnaire (BCKQ) will be utilised to understand patients' informational requirements and understanding and their knowledge base of COPD (29). The questionnaire is comprehensive and goes into detail, regarding

For peer review only - http://bmjopen.bmj.com/site/about/guidelines.xhtml

various aspects of COPD, for example epidemiology, signs and symptoms, and exacerbations and treatment.

Qualitative Data Collection

Those participants allocated to the intervention group are invited to take part in qualitative semi-structured focus groups at the end of the SPACE for COPD intervention. The aim of the focus groups will be to understand participants' experiences of the group-based self-management programme. More specifically, the data we collect will inform:

- Acceptability and usefulness of the programme to participants in this format and over this time period;
- The content of the intervention;
- Approaches to recruitment.

Up to 10 focus groups will be conducted with between 4-10 participants (number dependent on each intervention group size). Purposive sampling will be employed to recruit intervention participants. Audio-recorded focus group discussions (approximately 60 minutes) will be conducted face-to-face between each participant group, an experienced interviewer and an observer/note-taker. Focus groups will be transcribed verbatim by a professional transcriber, with identifiable information removed. Focus group questions have been devised based upon relevant literature and experience of the team.

Healthcare professionals delivering the SPACE for COPD© self-management support intervention will be invited to participate in a meeting to discuss and gain

For peer review only - http://bmjopen.bmj.com/site/about/guidelines.xhtml

insight into any potential facilitators/barriers to implementing the intervention in practice and derive practical recommendations for doing so. All staff involved in delivering the intervention will be approached to participate in a 2 hour discussion and will be given an information sheet and at least 48 hours to consider their involvement prior to consent. Minutes will be taken during the discussion and anonymised.

Data Analysis

Study Power

The power calculation was based on the primary outcome at 6-months (30, 31). To detect a mean ±SD between-group difference of 2.5±5.0 in the change in CAT with 80% power, 60 people per group are required (α=0.05, two-tailed). In anticipation of a possible 25% attrition rate, the total sample size was increased to 75 per group (150 in total).

Quantitative Analysis

This will primarily be completed on an intention-to-treat analysis. All quantitative data will be assessed for normality and analysed using appropriate parametric and non-parametric statistics, statistical significance will be set at p=0.05. Secondary per protocol analyses will be carried out.

For peer review only - http://bmjopen.bmj.com/site/about/guidelines.xhtml

A post hoc analysis will be carried out, which will exclude patients in either arm that received pulmonary rehabilitation as part of their usual care. We would anticipate that patients who participate within the study, will not require Pulmonary Rehabilitation. However, due to Pulmonary Rehabilitation being a part of 'best' usual care, this will not be withheld from the patient.

Quantitative data for all outcomes will be transcribed from the CRF onto an electronic database. A statistical software package will be used to carry out quantitative analyses. Predictive Analaytics Software (SPSS) will be used to analyse the data, the license for which is provided by University Hospitals of Leicester NHS Trust. Continuous variables will be presented as mean and standard deviation (SD) or median and inter quartile range (IQR), and categorical data will be presented as frequencies and percentages. Data will be checked for normality and appropriate parametric and nonparametric tests will be used. Any baseline differences will be adjusted for. Any missing data will be imputed, and both intention-to-treat and per protocol analyses will be conducted.

We have not secured funding for a healthcare utilisation analysis but would anticipate further CLAHRC (Collaboration and Leadership in Applied Health Research and Care) funding if the trial is clinically effective.

Qualitative Analysis

The focus groups will be analysed using Thematic Analysis (32) supported by Nvivo software (version 9). This approach follows six distinct stages: familiarization with

For peer review only - http://bmjopen.bmj.com/site/about/guidelines.xhtml

data; generating initial codes; searching for themes; reviewing themes; defining and naming themes and producing the report. The psychologist and the physiotherapist will carry out initial coding and a sample of interviews will be coded by another member of the team to ensure consistency and to enhance interpretive authenticity. Throughout the data analysis, the team will meet to discuss and review emerging themes and search for accounts that provide contesting views of the same phenomena or identify different phenomena. Our patient representatives will be invited to comment on our (anonymised) initial findings to ensure interpretations made by researchers stay close to the direct experience of patients (33).

All patient information that is collected during the course of the research will be kept strictly confidential. Any information about the patient which leaves the hospital will have their name and address removed. Participants will not be identified in any subsequent written material. Results will be reported in such a way that completely preserves confidentiality.

ETHICS AND DISSEMINATION

Ethics

The trial is sponsored by the University Hospitals of Leicester NHS Trust (study number 152408) and ethical approval was granted by the Hampshire B Research Ethics Committee (REC reference: 14/SC/11/69). Protocol amendments will be approved by the ethics committee and regulatory authorities as per current guidelines and will be communicated to relevant parties by the study team.

For peer review only - http://bmjopen.bmj.com/site/about/guidelines.xhtml

Dissemination

We plan to publish the results of the study in peer-reviewed journals and present them at appropriate national and international respiratory and physiotherapy conferences. Social media will be utilised to disseminate information and summaries of results to a wider population.

The CLAHRC East Midlands, is a large organisation which strives to improve health outcomes in the population across the East Midlands through delivering high quality, world class research. This organisation will be used to further disseminate results within the East Midlands. We also hope to provide a summary of results to the study participants. We also plan to hold a participant dissemination day towards the end of the study. This will enable participants to contribute to the final report and other result dissemination activities.

The institution also has an active and dynamic Public Involvement group for pulmonary and cardiovascular rehabilitation. The group will be used to create and co-ordinate strategies for further disseminating the results into the public domain.

The study may also be subject to internal and further external audits to ensure safety of the trial.

FUNDING

The research was funded by the National Institute for Health Research (NIHR) Collaboration for Leadership in Applied Health Research and Care East Midlands (CLAHRC EM), and took place at the University Hospitals of Leicester NHS Trust.

For peer review only - http://bmjopen.bmj.com/site/about/guidelines.xhtml

Support was also provided by the NIHR Leicester Respiratory Biomedical Research Unit (BRU). The views expressed are those of the authors and not necessarily those of the NHS, the NIHR or the Department of Health.

Roles of the funder: Management of staff, study progress, reporting and dissemination activities.

Study Sponsor

Carolyn Maloney. University Hospitals of Leicester NHS Trust, 0116 258 4109

Steering Committee and Acknowledgements

East Leicestershire CCG - Sue Price; Leicestershire Partnership Trust: Karen Moore, Alex Woodward, Gillian Doe; West Leicestershire CCG: Jake Cooke; Public Involvement: Patricia Overty, Freda Smart

AUTHORS CONTRIBUTIONS

SS is the principal investigator of the SPACE for COPD© study. KM, SS, SB, LA, LHW, SH and CB were involved in the development of the intervention and design of the trial. CB, SS, PK and THD have been involved in drafting the work or revising it critically for important intellectual content and have given the final approval of the version published.

COMPETING INTERESTS

None

For peer review only - http://bmjopen.bmj.com/site/about/guidelines.xhtml

Data sharing statement: Additional unpublished data from the study is still being collected and analysed and is only available to members of the study team.

For peer review only

For peer review only - http://bmjopen.bmj.com/site/about/guidelines.xhtml

Figure Legend:

Figure 1: Participant flow through the study

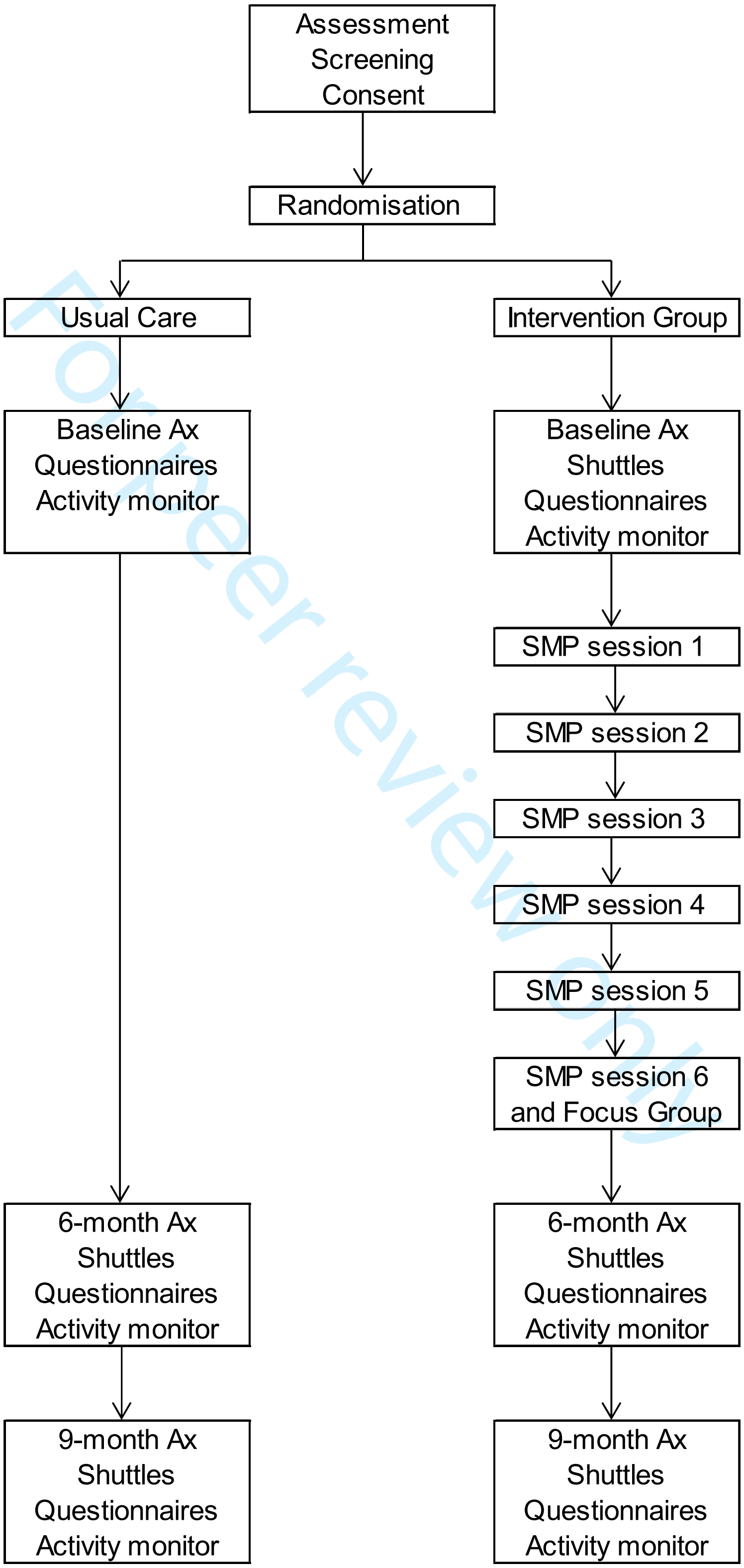

For peer review only - http://bmjopen.bmj.com/site/about/guidelines.xhtml

REFERENCES

(1) World Health Organisation. Chronic Obstructive Pulmonary Disease (COPD). 2016; Available at: http://www.who.int/respiratory/copd/en/. Accessed 05/06/2016.

(2) National Institute for Health and Care Excellence. Chronic obstructive pulmonary disease in over 16s: diagnosis and management. 2010;CG101.

(3) NHS England. Five year forward view. October 2014.

(4) Purdy S. Avoiding hospital admissions: What does the research evidence say? 2010.

(5) Zwerink M, Brusse-Keizer M, van der Valk PD, et al. Self management for patients with chronic obstructive pulmonary disease. Cochrane Database of Systematic Reviews .

(6) Effing T, Zielhuis G, Kerstjens H, et al. Community based physiotherapeutic exercise in COPD self-management: A randomised controlled trial. Respir Med 2011 3;105(3):418-426.

(7) Monninkhof E, van der Valk P, van der Palen J, et al. Effects of a comprehensive self-management programme in patients with chronic obstructive pulmonary disease. European Respiratory Journal 2003 European Respiratory Society;22(5):815-820.

(8) Bourbeau J, Julien M, Maltais F,et al. Reduction of hospital utilization in patients with chronic obstructive pulmonary disease: A disease-specific self-management intervention. Archives of Internal Medicine 2003 March 10;163(5):585-591.

For peer review only - http://bmjopen.bmj.com/site/about/guidelines.xhtml

(9) Apps LD, Mitchell KE, Harrison SL, et al. The development and pilot testing of the Self-management Programme of Activity, Coping and Education for Chronic Obstructive Pulmonary Disease (SPACE for COPD). International Journal of Chronic Obstructive Pulmonary Disease 2013;8:317-327.

(10) Mitchell KE, Johnson-Warrington V, Apps LD, et al. A self-management programme for COPD: a randomised controlled trial. European Respiratory Journal 2014 European Respiratory Society;44(6):1538-1547.

(11) Bandura A. Social Learning Theory. Englewood Cliffs, NJ: Prentice Hall; 1977.

(12) Bandura A. Self-efficacy: The exercise of control. New York: W.H. Freeman; 1997.

(13) Yalom I. Theory and practice of group psychotherapy. Fifth ed. New York: Basic Books; 2005.

(14) Sealed Envelope Ltd. Randomisation and and online databases for clinical trials. 2016; Available at: http://www.sealedenvelope.com/. Accessed 01/06, 2016.

(15) Pearce G, Parke HL, Pinnock H, et al. The PRISMS taxonomy of self-management support: derivation of a novel taxonomy and initial testing of its utility. Journal of Health Services Research & Policy 2016 April 01;21(2):73-82.

(16) Jones PW, Harding G, Berry P, et al. Development and first validation of the COPD Assessment Test. European Respiratory Journal 2009 European Respiratory Society;34(3):648-654.

For peer review only

(17) Singh SJ, Morgan M, Scott S, et al. Development of a shuttle walking test of disability in patients with chronic airways obstruction. Thorax 1992;47(12):1019-1024.

(18) Singh SJ, Jones PW, Evans R, et al. Minimum clinically important improvement for the incremental shuttle walking test. Thorax 2008 September 01;63(9):775-777.

(19) Revill SM, Morgan MDL, Singh SJ, et al. The endurance shuttle walk: a new field test for the assessment of endurance capacity in chronic obstructive pulmonary disease. Thorax 1999 March 01;54(3):213-222.

(20) EuroQol Group. EuroQol - a new facility for the measurement of health-related quality of life. Health Policy 1990;16(3):199-208.

(21) Rutten-van-Molken MP, Oostenbrink JB, Tashkin DP, et al. Does Quality of Life of COPD Patients as Measured by the Generic EuroQol Five-Dimension Questionnaire Differentiate Between COPD Severity Stages? Chest 2006 October;130(4):1117-1128.

(22) Williams JEA, Singh SJ, Sewell L, et al. Development of a self-reported Chronic Respiratory Questionnaire (CRQ-SR). Thorax 2001 December 01;56(12):954-959.

(23) Juniper EF, Guyatt GH, Willan A, et al. Determining a minimal important change in a disease-specific quality of life questionnaire. J Clin Epidemiol 1994 January 1994;47(1):81-87.

(24) Zigmond AS, Snaith RP. The Hospital Anxiety and Depression Scale. Acta Psychiatr Scand 1983;67(6):361-370.

For peer review only - http://bmjopen.bmj.com/site/about/guidelines.xhtml

(25) Al-Gamal E, Yorke J. Perceived breathlessness and psychological distress among patients with chronic obstructive pulmonary disease and their spouses. Nurs Health Sci 2014;16(1):103-111.

(26) Bjelland I, Dahl A, Haug T, et al. The validity of the hospital anxiety and depression scale: An updated literature review. Journal of Psychosomatic Research 2002;52(2):69-77.

(27) Hung C, Liu C, Wang S, et al. The cut-off points of the Depression and Somatic Symptoms Scale and the Hospital Anxiety and Depression Scale in detecting non-full remission and a current major depressive episode. Int J Psychiatry Clin Pract 2012 03/01;16(1):33-40.

(28) Donald M, Ware RS, Ozolins IZ, et al. The role of patient activation in frequent attendance at primary care: A population-based study of people with chronic disease. Patient Educ Couns 2011 5;83(2):217-221.

(29) White R, Walker P, Roberts S, et al. Bristol COPD Knowledge Questionnaire (BCKQ): testing what we teach patients about COPD. Chronic Respiratory Disease 2006 July 01;3(3):123-131.

(30) Jones PW, Harding G, Wiklund I, et al. Tests of the responsiveness of the copd assessment test following acute exacerbation and pulmonary rehabilitation. Chest 2012 July 1;142(1):134-140.

(31) Kon SSC, Canavan JL, Jones SE, et al. Minimum clinically important difference for the COPD Assessment Test: a prospective analysis. The Lancet Respiratory Medicine 2014 3;2(3):195-203.

For peer review only - http://bmjopen.bmj.com/site/about/guidelines.xhtml

For peer review only

(32) Braun V, Clarke V. Using thematic analysis in psychology. Qualitative Research in Psychology 2006;3(2):77-101.

(33) Boote J, Baird W, Beecroft C. Public involvement at the design stage of primary health research: A narrative review of case examples. Health Policy 2010 4;95(1):10-23.

For peer review only

For peer review only - http://bmjopen.bmj.com/site/about/guidelines.xhtml

SPIRIT
STANDARD PROTOCOL ITEMS: RECOMMENDATIONS FOR INTERVENTIONAL TRIALS

SPIRIT 2013 Checklist: Recommended items to address in a clinical trial protocol and related documents*

Section/item	Item No	Description	Addressed on page number
**Administrative information**			
Title	1	Descriptive title identifying the study design, population, interventions, and, if applicable, trial acronym	1
Trial registration	2a	Trial identifier and registry name. If not yet registered, name of intended registry	3
	2b	All items from the World Health Organization Trial Registration Data Set	N/A
Protocol version	3	Date and version identifier	3
Funding	4	Sources and types of financial, material, and other support	27 and 28
Roles and responsibilities	5a	Names, affiliations, and roles of protocol contributors	1
	5b	Name and contact information for the trial sponsor	28
	5c	Role of study sponsor and funders, if any, in study design; collection, management, analysis, and interpretation of data; writing of the report; and the decision to submit the report for publication, including whether they will have ultimate authority over any of these activities	28
	5d	Composition, roles, and responsibilities of the coordinating centre, steering committee, endpoint adjudication committee, data management team, and other individuals or groups overseeing the trial, if applicable (see Item 21a for data monitoring committee)	28

For peer review only

For peer review only - http://bmjopen.bmj.com/site/about/guidelines.xhtml

**Introduction**			
Background and rationale	6a	Description of research question and justification for undertaking the trial, including summary of relevant studies (published and unpublished) examining benefits and harms for each intervention	5-8
	6b	Explanation for choice of comparators	8
Objectives	7	Specific objectives or hypotheses	8
Trial design	8	Description of trial design including type of trial (eg, parallel group, crossover, factorial, single group), allocation ratio, and framework (eg, superiority, equivalence, noninferiority, exploratory)	9
**Methods: Participants, interventions, and outcomes**			
Study setting	9	Description of study settings (eg, community clinic, academic hospital) and list of countries where data will be collected. Reference to where list of study sites can be obtained	9
Eligibility criteria	10	Inclusion and exclusion criteria for participants. If applicable, eligibility criteria for study centres and individuals who will perform the interventions (eg, surgeons, psychotherapists)	10
Interventions	11a	Interventions for each group with sufficient detail to allow replication, including how and when they will be administered	11-16
	11b	Criteria for discontinuing or modifying allocated interventions for a given trial participant (eg, drug dose change in response to harms, participant request, or improving/worsening disease)	N/A
	11c	Strategies to improve adherence to intervention protocols, and any procedures for monitoring adherence (eg, drug tablet return, laboratory tests)	17
	11d	Relevant concomitant care and interventions that are permitted or prohibited during the trial	11
Outcomes	12	Primary, secondary, and other outcomes, including the specific measurement variable (eg, systolic blood pressure), analysis metric (eg, change from baseline, final value, time to event), method of aggregation (eg, median, proportion), and time point for each outcome. Explanation of the clinical relevance of chosen efficacy and harm outcomes is strongly recommended	18-24
Participant timeline	13	Time schedule of enrolment, interventions (including any run-ins and washouts), assessments, and visits for participants. A schematic diagram is highly recommended (see Figure)	19 & diagram 1

For peer review only - http://bmjopen.bmj.com/site/about/guidelines.xhtml

Sample size	14	Estimated number of participants needed to achieve study objectives and how it was determined, including clinical and statistical assumptions supporting any sample size calculations	24
Recruitment	15	Strategies for achieving adequate participant enrolment to reach target sample size	9-10
**Methods: Assignment of interventions (for controlled trials)**			
Allocation:			
Sequence generation	16a	Method of generating the allocation sequence (eg, computer-generated random numbers), and list of any factors for stratification. To reduce predictability of a random sequence, details of any planned restriction (eg, blocking) should be provided in a separate document that is unavailable to those who enrol participants or assign interventions	10-11
Allocation concealment mechanism	16b	Mechanism of implementing the allocation sequence (eg, central telephone; sequentially numbered, opaque, sealed envelopes), describing any steps to conceal the sequence until interventions are assigned	10
Implementation	16c	Who will generate the allocation sequence, who will enrol participants, and who will assign participants to interventions	10-11
Blinding (masking)	17a	Who will be blinded after assignment to interventions (eg, trial participants, care providers, outcome assessors, data analysts), and how	11 & 19
	17b	If blinded, circumstances under which unblinding is permissible, and procedure for revealing a participant's allocated intervention during the trial	11
**Methods: Data collection, management, and analysis**			
Data collection methods	18a	Plans for assessment and collection of outcome, baseline, and other trial data, including any related processes to promote data quality (eg, duplicate measurements, training of assessors) and a description of study instruments (eg, questionnaires, laboratory tests) along with their reliability and validity, if known. Reference to where data collection forms can be found, if not in the protocol	18-24
	18b	Plans to promote participant retention and complete follow-up, including list of any outcome data to be collected for participants who discontinue or deviate from intervention protocols	N/A

For peer review only - http://bmjopen.bmj.com/site/about/guidelines.xhtml

Data management	19	Plans for data entry, coding, security, and storage, including any related processes to promote data quality (eg, double data entry; range checks for data values). Reference to where details of data management procedures can be found, if not in the protocol	24-26
Statistical methods	20a	Statistical methods for analysing primary and secondary outcomes. Reference to where other details of the statistical analysis plan can be found, if not in the protocol	25
	20b	Methods for any additional analyses (eg, subgroup and adjusted analyses)	25
	20c	Definition of analysis population relating to protocol non-adherence (eg, as randomised analysis), and any statistical methods to handle missing data (eg, multiple imputation)	25
**Methods: Monitoring**			
Data monitoring	21a	Composition of data monitoring committee (DMC); summary of its role and reporting structure; statement of whether it is independent from the sponsor and competing interests; and reference to where further details about its charter can be found, if not in the protocol. Alternatively, an explanation of why a DMC is not needed	N/A
	21b	Description of any interim analyses and stopping guidelines, including who will have access to these interim results and make the final decision to terminate the trial	N/A
Harms	22	Plans for collecting, assessing, reporting, and managing solicited and spontaneously reported adverse events and other unintended effects of trial interventions or trial conduct	18
Auditing	23	Frequency and procedures for auditing trial conduct, if any, and whether the process will be independent from investigators and the sponsor	N/A
**Ethics and dissemination**			
Research ethics approval	24	Plans for seeking research ethics committee/institutional review board (REC/IRB) approval	26
Protocol amendments	25	Plans for communicating important protocol modifications (eg, changes to eligibility criteria, outcomes, analyses) to relevant parties (eg, investigators, REC/IRBs, trial participants, trial registries, journals, regulators)	26

For peer review only - http://bmjopen.bmj.com/site/about/guidelines.xhtml

Consent or assent	26a	Who will obtain informed consent or assent from potential trial participants or authorised surrogates, and how (see Item 32)	10 & 19
	26b	Additional consent provisions for collection and use of participant data and biological specimens in ancillary studies, if applicable	N/A
Confidentiality	27	How personal information about potential and enrolled participants will be collected, shared, and maintained in order to protect confidentiality before, during, and after the trial	26
Declaration of interests	28	Financial and other competing interests for principal investigators for the overall trial and each study site	28
Access to data	29	Statement of who will have access to the final trial dataset, and disclosure of contractual agreements that limit such access for investigators	29
Ancillary and post-trial care	30	Provisions, if any, for ancillary and post-trial care, and for compensation to those who suffer harm from trial participation	N/A
Dissemination policy	31a	Plans for investigators and sponsor to communicate trial results to participants, healthcare professionals, the public, and other relevant groups (eg, via publication, reporting in results databases, or other data sharing arrangements), including any publication restrictions	27
	31b	Authorship eligibility guidelines and any intended use of professional writers	N/A
	31c	Plans, if any, for granting public access to the full protocol, participant-level dataset, and statistical code	N/A
**Appendices**			
Informed consent materials	32	Model consent form and other related documentation given to participants and authorised surrogates	PIS and consent form attached separately
Biological specimens	33	Plans for collection, laboratory evaluation, and storage of biological specimens for genetic or molecular analysis in the current trial and for future use in ancillary studies, if applicable	N/A

*It is strongly recommended that this checklist be read in conjunction with the SPIRIT 2013 Explanation & Elaboration for important clarification on the items. Amendments to the protocol should be tracked and dated. The SPIRIT checklist is copyrighted by the SPIRIT Group under the Creative Commons "Attribution-NonCommercial-NoDerivs 3.0 Unported" license.

For peer review only - http://bmjopen.bmj.com/site/about/guidelines.xhtml

BMJ Open

A self-management programme of activity coping and education - SPACE FOR COPD© - in primary care: The protocol for a pragmatic trial

Journal:	*BMJ Open*
Manuscript ID	bmjopen-2016-014463.R1
Article Type:	Protocol
Date Submitted by the Author:	24-Feb-2017
Complete List of Authors:	Bourne, Claire; University Hospitals of Leicester NHS Trust, Centre of Exercise and Rehabilitation Science<br>Kanabar, Pratiksha; University Hospitals of Leicester NHS Trust, Centre of Exercise and Rehabilitation Science, Respiratory Biomedical Research Unit, Glenfield Hospital<br>Mitchell, Katy; University Hospitals of Leicester NHS Trust, Centre of Exercise and Rehabilitation Science, Respiratory Biomedical Research Unit, Glenfield Hospital<br>Schreder, Sally; University Hospitals of Leicester NHS Trust, Centre of Exercise and Rehabilitation Science; University of Leicester Diabetes Research Centre, Air Zone<br>Houchen-Wolloff, Linzy; University Hospitals of Leicester NHS Trust, Centre of Exercise and Rehabilitation Science, Respiratory Biomedical Research Unit, Glenfield Hospital<br>Bankart, M. John; Keele University, Institute of Primary Care<br>Apps, Lindsay ; Leicester Respiratory Biomedical Research Unit, Centre for Exercise and Rehabilitation Science, Respiratory Biomedical Research Unit, Glenfield Hospital<br>Hewitt, Stacey; University Hospitals of Leicester NHS Trust, Centre of Exercise and Rehabilitation Science, Leicester Respiratory Biomedical Research Unit, Respiratory Biomedical Research Unit, Glenfield Hospital<br>Harvey-Dunstan, Theresa; University Hospitals of Leicester NHS Trust, Centre of Exercise and Rehabilitation Science, Leicester Respiratory Biomedical Research Unit, Respiratory Biomedical Research Unit, Glenfield Hospital<br>Singh, Sally; University Hospitals of Leicester NHS Trust, Cardiac/Pulmonary Rehabilitation; Loughborough University, National Centre for Sport and Exercise Medicine
**Primary Subject Heading**:	Respiratory medicine
Secondary Subject Heading:	Patient-centred medicine
Keywords:	COPD, Self-management support, PRIMARY CARE

For peer review only

For peer review only - http://bmjopen.bmj.com/site/about/guidelines.xhtml

University Hospitals of Leicester NHS
NHS Trust

Glenfield Hospital
Groby Road
Leicester
LE3 9QP

Tel: 0116 287 1471 ext 250 2762
or 250 2758
Fax: 0116 258 3950
Minicom: 0116 287 9852

A self-management programme of activity coping and education - SPACE FOR COPD - in primary care: a pragmatic trial (Version 5 08/05/2015)

You are being invited to take part in research study being conducted by University Hospitals of Leicester NHS Trust. Before you decide whether or not to take part it is important for you to understand why the research is being done and what it will involve. Please take time to read the following information carefully and discuss it with others if you wish. Ask us if there is anything that is not clear or if you would like more information. Take time to decide whether or not you wish to take part.

We have developed a self-management manual for patients with Chronic Obstructive Pulmonary Disease (COPD). This has been done with enormous help from patients like yourself. The manual covers issues such as drug and symptom management, exercise and nutrition at home.

In a previous study, we provided this manual for people to work through at home, with one initial meeting with a healthcare professional. While that study showed there were several benefits to having the manual, we would now like to see if the benefits will last longer if support from the healthcare professional is increased. Also patients have told us they would like the manual to be and supported in a peer group environment. This study, therefore, will test the effectiveness of using the manual within a group setting over a number of sessions.

We would like to know how effective the use of this manual may be when delivered and supported in a group environment. This will help us improve future patient care.

2. Why have I been invited?

As an individual with COPD you have been identified as a suitable participant by your GP or your healthcare professional. You might have also been involved in a previous research trial and agreed to be contacted again or agreed to be contacted about future trials for which you might be eligible. It is important we see how people progress using the manual we have developed compared to those who receive standard care from their GP or healthcare professional. This knowledge may help us develop and improve future services.

3. Do I have to take part?

It is up to you to decide whether or not to take part. If you do decide to take part you will be given this information sheet to keep and be asked to sign a consent form. If you decide to

For peer review only - http://bmjopen.bmj.com/site/about/guidelines.xhtml

take part you are still free to withdraw at any time and without giving a reason. Your decision to withdraw will not influence any medical care you receive.

4. If you agree to part what will happen?

Once you have provisionally agreed to take part in the research you will be contacted to arrange a date and time to discuss the project in more detail. You will be invited to attend an appointment which will take place at Glenfield Hospital. If you still wish to go ahead at this stage then we will ask you to sign a consent form to take part in the study. Following this we will do some lung function tests. These are tests where you blow out into a tube as hard as you can. This confirms that you do have a diagnosis of COPD. You will then be randomly allocated to one of two arms of the trial. Half of the people in the study will be randomly allocated to receive the self-management group programme. The other half will be randomly assigned to 'usual care' which means that we will not change any of your current treatment or care. You will be randomly allocated to one of these two arms of the study by a computer-based system. This means that we will not know before hand which arm of the study you will be in. It is important that this is done at random in order to keep the results of the study fair.

5. What do I have to do?

If you are assigned to the usual care group you will continue as normal with the treatment you receive from your GP and healthcare team. At your first visit with us, you will be requested to complete some questionnaires about your health status. These will take about 20 minutes to complete. We will also ask you to wear an activity monitor for the following 7 days at home. The monitor is a small device which is worn around your arm, and it measures your activity levels at home. We will arrange to collect this from you after you have finished wearing it. We will ask you to repeat the questionnaires and wearing of the activity monitor after 6 months, and then again 3 months later (9 months from starting the study and your final assessment). In addition to the questionnaires and activity monitor at 6 months and 9 months, we will ask you to attend the hospital to do some walking tests. These will be done at Glenfield Hospital. There will usually be three walking tests, which are done on a flat corridor, walking up and down a 10 metre course. It is usual to become breathless during these tests, however we will only ask you to do as much as you feel you can.

If you are assigned to the self-management group you will undergo the same assessments at the same time points as the usual care group. However, in addition to these we will also ask you to do the walking tests at all three time points (i.e. your first visit, 6 month visit and 9 month visit). These will all take place at Glenfield Hospital. In between your first visit and your 6 month visit we will ask you to attend the group sessions to introduce you to the self-management programme. These will take place at a community centre. There will be approximately between six and 10 people in each group, and all sessions will be led by 2 healthcare professionals. Each session will last approximately two hours. At your first session you will be given the SPACE FOR COPD manual, and each session thereafter will discuss a different topic about how to manage with COPD. You will be advised how you can increase your activities at home, improve management of your breathlessness and learn what to do if you develop a chest infection. You do not have to discuss anything within the group if you do not wish to, and everything discussed within the group will be kept confidential. At the end of the self-management programme we will also ask you to take part in a focus group discussion where you will have an opportunity to discuss your experiences of the programme and whether you thought it was useful to you. The focus group will last

For peer review only - http://bmjopen.bmj.com/site/about/guidelines.xhtml

approximately 1 hour. The discussions will be audio recorded and transcribed for research purposes.

During the study, and immediately afterwards the study team will access your medical notes to obtain long term follow up data (for example GP visits, hospital admissions).

6. Are there any risks, disadvantages or side-effects of taking part?

We do not expect there are any risks to taking part in this study.

One potential disadvantage to taking part is the time you give to the study. Your first visit may take up to two hours. Your six-month and nine-month visits may take up to one hour each.

Whilst completing the walking tests, you may become short of breath, however we expect this will recover once you sit and rest. Afterwards, you may experience some achiness in your muscles and feel tired. This is usually only lasts a day or two.

7. What are the possible benefits of taking part?

We hope that the research will aid you in your understanding of COPD and how you can improve your symptoms. Information gathered will inform both present and future research, aiming to provide better care for people living with COPD.

8. What if something goes wrong?

If you are harmed by taking part in this research project, there are no special compensation arrangements. If you are harmed due to someone's negligence, then you may have grounds for a legal action but you may have to pay for it. Regardless of this, if you wish to complain, or have any concerns about any aspect of the way you have been approached or treated during the course of this study, the normal National Health Service complaints mechanisms would be available to you.

Should you wish to discuss any concerns about this study further, the Patient Information and Liaison Service is a point of contact for you. Their telephone number is 0808 178 8337.

9. Will my taking part in this study be kept confidential?

All information that is collected about you during the course of the research will be kept strictly confidential. Any information about you, which leaves the medical centre, will have your name and address removed so that you cannot be recognised from it. Some of your anonymised data will be shared with Insignia Health (a healthcare organisation) and entered onto a secure database to help improve patient outcome measures. This will be basic information, for example age, gender and some questionnaire data. Participants will not be identified in any subsequent written material. Results will be reported in such a way that completely preserves confidentiality. Data will be stored at Glenfield Hospital, and on University Hospitals of Leicester secure networks.

For peer review only - http://bmjopen.bmj.com/site/about/guidelines.xhtml

We will, with your consent inform your GP of your participation in the trial and share with your GP any relevant test results that have been made during your participation in the trial.

10. What will happen to the results of the research study?

The results of the study will be written in peer and lay journals, professional publications and presentations made at relevant conferences. Results will be reported in such a way that preserves confidentiality. All participants will also receive a summary of the results.

11. Who is organising and funding the research?

This study is being organised by staff employed at University Hospitals of Leicester NHS trust. It is being funded by a Collaboration and Leadership in Applied Health Research and Care East Midlands (CLAHRC).

12. Who has reviewed the study?

All research that involves the NHS is reviewed by an NHS Research Ethics Committee. This study has been reviewed and given a favourable opinion by Hampshire B research Ethics Committee. This does not guarantee that you will not come to any harm if you take part. However, approval means that the committee is satisfied that your rights will be respected, that any risks have been reduced to a minimum and balanced against possible benefits and that you have been given sufficient information of which to make an informed decision. The study has also been reviewed and is sponsored by University Hospitals of Leicester.

13. Contact for further information

If you have any concerns or other questions about this study or the way it has been carried out, you should contact the principal researcher Professor Sally Singh:

Contact for further information:

The research team
Centre for Exercise and Rehabilitation Science
Leicester Respiratory Biomedical Research Unit,
Glenfield Hospital,
Leicester,
LE3 9PQ

0116 2583035
sally.singh@uhl-tr.nhs.uk

Thank you for reading this
Yours faithfully

Sally Singh

For peer review only

For peer review only - http://bmjopen.bmj.com/site/about/guidelines.xhtml

University Hospitals of Leicester NHS Trust

Glenfield Hospital
Groby Road
Leicester
LE3 9QP

Tel: 0116 287 1471
Fax: 0116 258 3950
Minicom: 0116 287 9852

CONSENT FORM for Patients
(Version 5 08/05/2015)

Identification Number for this study:

A self-management programme of activity coping and education - SPACE FOR COPD - in primary care: a pragmatic trial

Please initial box

1. I confirm that I have read and understand the information sheet version 5 (08/05/2015) for the above study and have had the opportunity to ask questions. ☐
2. I understand that my participation is voluntary and that I am free to withdraw at any time, without giving any reason, without my medical care or legal rights being affected. ☐
3. I understand that the research team will access my medical notes to obtain data that is relevant to this study, such as information about healthcare use and to follow up information about my health. I give the researchers' permission to access my medical notes for this purpose. ☐
4. I understand that my personal information may be stored, safely and securely, at University Hospitals of Leicester as well as at my GP practice. ☐
5. I agree for my GP to be informed of my participation and that results from some of the tests performed as part of this study may be sent to my GP. ☐
6. I understand that some of my data will be shared with Insignia Health and entered onto a secure database to help improve patient outcome measures, if it is anonymised. ☐
7. I agree to take part in the above study. ☐
8. I understand that all discussions at group sessions are confidential and I agree to respect this confidentiality. ☐
9. I agree to take part in a focus group discussion at the end of the self-management programme which will be recorded and transcribed for research purposes. I also agree that anonymised direct quotes can be used for research purposes**.** ☐ yes ☐ no

Name of Patient	Date	Signature
Name of Person taking consent	Date	Signature

For peer review only - http://bmjopen.bmj.com/site/about/guidelines.xhtml

SPIRIT
STANDARD PROTOCOL ITEMS: RECOMMENDATIONS FOR INTERVENTIONAL TRIALS

SPIRIT 2013 Checklist: Recommended items to address in a clinical trial protocol and related documents*

Section/item	Item No	Description	Addressed on page number
**Administrative information**			
Title	1	Descriptive title identifying the study design, population, interventions, and, if applicable, trial acronym	1
Trial registration	2a	Trial identifier and registry name. If not yet registered, name of intended registry	3
	2b	All items from the World Health Organization Trial Registration Data Set	N/A
Protocol version	3	Date and version identifier	3
Funding	4	Sources and types of financial, material, and other support	27 and 28
Roles and responsibilities	5a	Names, affiliations, and roles of protocol contributors	1
	5b	Name and contact information for the trial sponsor	28
	5c	Role of study sponsor and funders, if any, in study design; collection, management, analysis, and interpretation of data; writing of the report; and the decision to submit the report for publication, including whether they will have ultimate authority over any of these activities	28
	5d	Composition, roles, and responsibilities of the coordinating centre, steering committee, endpoint adjudication committee, data management team, and other individuals or groups overseeing the trial, if applicable (see Item 21a for data monitoring committee)	28

For peer review only - http://bmjopen.bmj.com/site/about/guidelines.xhtml

**Introduction**			
Background and rationale	6a	Description of research question and justification for undertaking the trial, including summary of relevant studies (published and unpublished) examining benefits and harms for each intervention	5-8
	6b	Explanation for choice of comparators	8
Objectives	7	Specific objectives or hypotheses	8
Trial design	8	Description of trial design including type of trial (eg, parallel group, crossover, factorial, single group), allocation ratio, and framework (eg, superiority, equivalence, noninferiority, exploratory)	9
**Methods: Participants, interventions, and outcomes**			
Study setting	9	Description of study settings (eg, community clinic, academic hospital) and list of countries where data will be collected. Reference to where list of study sites can be obtained	9
Eligibility criteria	10	Inclusion and exclusion criteria for participants. If applicable, eligibility criteria for study centres and individuals who will perform the interventions (eg, surgeons, psychotherapists)	10
Interventions	11a	Interventions for each group with sufficient detail to allow replication, including how and when they will be administered	11-16
	11b	Criteria for discontinuing or modifying allocated interventions for a given trial participant (eg, drug dose change in response to harms, participant request, or improving/worsening disease)	N/A
	11c	Strategies to improve adherence to intervention protocols, and any procedures for monitoring adherence (eg, drug tablet return, laboratory tests)	17
	11d	Relevant concomitant care and interventions that are permitted or prohibited during the trial	11
Outcomes	12	Primary, secondary, and other outcomes, including the specific measurement variable (eg, systolic blood pressure), analysis metric (eg, change from baseline, final value, time to event), method of aggregation (eg, median, proportion), and time point for each outcome. Explanation of the clinical relevance of chosen efficacy and harm outcomes is strongly recommended	18-24
Participant timeline	13	Time schedule of enrolment, interventions (including any run-ins and washouts), assessments, and visits for participants. A schematic diagram is highly recommended (see Figure)	19 & diagram 1

For peer review only - http://bmjopen.bmj.com/site/about/guidelines.xhtml

Sample size	14	Estimated number of participants needed to achieve study objectives and how it was determined, including clinical and statistical assumptions supporting any sample size calculations	24
Recruitment	15	Strategies for achieving adequate participant enrolment to reach target sample size	9-10
**Methods: Assignment of interventions (for controlled trials)**			
Allocation:			
Sequence generation	16a	Method of generating the allocation sequence (eg, computer-generated random numbers), and list of any factors for stratification. To reduce predictability of a random sequence, details of any planned restriction (eg, blocking) should be provided in a separate document that is unavailable to those who enrol participants or assign interventions	10-11
Allocation concealment mechanism	16b	Mechanism of implementing the allocation sequence (eg, central telephone; sequentially numbered, opaque, sealed envelopes), describing any steps to conceal the sequence until interventions are assigned	10
Implementation	16c	Who will generate the allocation sequence, who will enrol participants, and who will assign participants to interventions	10-11
Blinding (masking)	17a	Who will be blinded after assignment to interventions (eg, trial participants, care providers, outcome assessors, data analysts), and how	11 & 19
	17b	If blinded, circumstances under which unblinding is permissible, and procedure for revealing a participant's allocated intervention during the trial	11
**Methods: Data collection, management, and analysis**			
Data collection methods	18a	Plans for assessment and collection of outcome, baseline, and other trial data, including any related processes to promote data quality (eg, duplicate measurements, training of assessors) and a description of study instruments (eg, questionnaires, laboratory tests) along with their reliability and validity, if known. Reference to where data collection forms can be found, if not in the protocol	18-24
	18b	Plans to promote participant retention and complete follow-up, including list of any outcome data to be collected for participants who discontinue or deviate from intervention protocols	N/A

For peer review only - http://bmjopen.bmj.com/site/about/guidelines.xhtml

Data management	19	Plans for data entry, coding, security, and storage, including any related processes to promote data quality (eg, double data entry; range checks for data values). Reference to where details of data management procedures can be found, if not in the protocol	24-26
Statistical methods	20a	Statistical methods for analysing primary and secondary outcomes. Reference to where other details of the statistical analysis plan can be found, if not in the protocol	25
	20b	Methods for any additional analyses (eg, subgroup and adjusted analyses)	25
	20c	Definition of analysis population relating to protocol non-adherence (eg, as randomised analysis), and any statistical methods to handle missing data (eg, multiple imputation)	25
**Methods: Monitoring**			
Data monitoring	21a	Composition of data monitoring committee (DMC); summary of its role and reporting structure; statement of whether it is independent from the sponsor and competing interests; and reference to where further details about its charter can be found, if not in the protocol. Alternatively, an explanation of why a DMC is not needed	N/A
	21b	Description of any interim analyses and stopping guidelines, including who will have access to these interim results and make the final decision to terminate the trial	N/A
Harms	22	Plans for collecting, assessing, reporting, and managing solicited and spontaneously reported adverse events and other unintended effects of trial interventions or trial conduct	18
Auditing	23	Frequency and procedures for auditing trial conduct, if any, and whether the process will be independent from investigators and the sponsor	N/A
**Ethics and dissemination**			
Research ethics approval	24	Plans for seeking research ethics committee/institutional review board (REC/IRB) approval	26
Protocol amendments	25	Plans for communicating important protocol modifications (eg, changes to eligibility criteria, outcomes, analyses) to relevant parties (eg, investigators, REC/IRBs, trial participants, trial registries, journals, regulators)	26

For peer review only - http://bmjopen.bmj.com/site/about/guidelines.xhtml

Consent or assent	26a	Who will obtain informed consent or assent from potential trial participants or authorised surrogates, and how (see Item 32)	10 & 19
	26b	Additional consent provisions for collection and use of participant data and biological specimens in ancillary studies, if applicable	N/A
Confidentiality	27	How personal information about potential and enrolled participants will be collected, shared, and maintained in order to protect confidentiality before, during, and after the trial	26
Declaration of interests	28	Financial and other competing interests for principal investigators for the overall trial and each study site	28
Access to data	29	Statement of who will have access to the final trial dataset, and disclosure of contractual agreements that limit such access for investigators	29
Ancillary and post-trial care	30	Provisions, if any, for ancillary and post-trial care, and for compensation to those who suffer harm from trial participation	N/A
Dissemination policy	31a	Plans for investigators and sponsor to communicate trial results to participants, healthcare professionals, the public, and other relevant groups (eg, via publication, reporting in results databases, or other data sharing arrangements), including any publication restrictions	27
	31b	Authorship eligibility guidelines and any intended use of professional writers	N/A
	31c	Plans, if any, for granting public access to the full protocol, participant-level dataset, and statistical code	N/A
**Appendices**			
Informed consent materials	32	Model consent form and other related documentation given to participants and authorised surrogates	PIS and consent form attached separately
Biological specimens	33	Plans for collection, laboratory evaluation, and storage of biological specimens for genetic or molecular analysis in the current trial and for future use in ancillary studies, if applicable	N/A

*It is strongly recommended that this checklist be read in conjunction with the SPIRIT 2013 Explanation & Elaboration for important clarification on the items. Amendments to the protocol should be tracked and dated. The SPIRIT checklist is copyrighted by the SPIRIT Group under the Creative Commons "Attribution-NonCommercial-NoDerivs 3.0 Unported" license.

For peer review only - http://bmjopen.bmj.com/site/about/guidelines.xhtml

BMJ Open

A self-management programme of activity coping and education - SPACE FOR COPD© - in primary care: The protocol for a pragmatic trial

Journal:	*BMJ Open*
Manuscript ID	bmjopen-2016-014463.R2
Article Type:	Protocol
Date Submitted by the Author:	24-Apr-2017
Complete List of Authors:	Bourne, Claire; University Hospitals of Leicester NHS Trust, Centre of Exercise and Rehabilitation Science<br>Kanabar, Pratiksha; University Hospitals of Leicester NHS Trust, Centre of Exercise and Rehabilitation Science, Respiratory Biomedical Research Unit, Glenfield Hospital<br>Mitchell, Katy; University Hospitals of Leicester NHS Trust, Centre of Exercise and Rehabilitation Science, Respiratory Biomedical Research Unit, Glenfield Hospital<br>Schreder, Sally; University Hospitals of Leicester NHS Trust, Centre of Exercise and Rehabilitation Science; University of Leicester Diabetes Research Centre, Air Zone<br>Houchen-Wolloff, Linzy; University Hospitals of Leicester NHS Trust, Centre of Exercise and Rehabilitation Science, Respiratory Biomedical Research Unit, Glenfield Hospital<br>Bankart, M. John; Keele University, Institute of Primary Care<br>Apps, Lindsay ; Leicester Respiratory Biomedical Research Unit, Centre for Exercise and Rehabilitation Science, Respiratory Biomedical Research Unit, Glenfield Hospital<br>Hewitt, Stacey; University Hospitals of Leicester NHS Trust, Centre of Exercise and Rehabilitation Science, Leicester Respiratory Biomedical Research Unit, Respiratory Biomedical Research Unit, Glenfield Hospital<br>Harvey-Dunstan, Theresa; University Hospitals of Leicester NHS Trust, Centre of Exercise and Rehabilitation Science, Leicester Respiratory Biomedical Research Unit, Respiratory Biomedical Research Unit, Glenfield Hospital<br>Singh, Sally; University Hospitals of Leicester NHS Trust, Cardiac/Pulmonary Rehabilitation; Loughborough University, National Centre for Sport and Exercise Medicine
**Primary Subject Heading**:	Respiratory medicine
Secondary Subject Heading:	Patient-centred medicine
Keywords:	COPD, Self-management support, PRIMARY CARE

For peer review only

A self-management programme of activity coping and education - SPACE FOR COPD© - in primary care: The protocol for a pragmatic trial

C. Bourne[1],P. Kanabar[1], K. Mitchell[1], S. Schreder[1], L. Houchen-Wolloff[1], J. Bankart[2], L. Apps[1], S. Hewitt[1], T. Harvey-Dunstan[1], S. Singh[1, 3]

[1]Collaboration and Leadership for Applied Health Research and Care - East Midlands, Centre of Exercise and Rehabilitation Science, Respiratory Biomedical Research Unit, Glenfield hospital, Groby Road, Leicester, Leicestershire, UK, LE3 9QP

[2]Department of Primary Care and Health Sciences, Keele University, Keele, UK

[3]National Centre for Sport and Exercise Medicine, Loughborough University, Leicestershire, UK, LE11 3TU

Corresponding Author:

Dr Claire Bourne

Centre of Exercise and Rehabilitation Science,

Respiratory Biomedical Research Unit,

Glenfield hospital,

Groby Road,

LE3 9QP

Email: Claire.Bourne@uhl-tr.nhs.uk

Tel: 0116 258 3035

Key words: COPD, self-management support, primary care

Word count: 3,906 words

For peer review only

ABSTRACT

Introduction

National guidance for COPD suggests that self-management support be provided for patients. Our institution has developed a standardised, manual-based, supported self-management programme: Self-Management Programme of Activity Coping and Education - SPACE for COPD©. SPACE was previously piloted on a 1-2-1 basis, delivered by researchers, to individuals with COPD. Discussions with stakeholders highlighted considerable interest in delivering the SPACE FOR COPD© intervention as a group-based self-management programme facilitated by healthcare professionals (HCPs) in primary care settings. The study aims are to explore the feasibility, acceptability and efficacy for the intervention to be delivered and supported by Healthcare Professionals (HCP's); and to examine whether group-based delivery of SPACE FOR COPD©, with sustained support, improves patient outcomes following the SPACE FOR COPD© intervention.

Methods and Analysis

A prospective, multi-site, single-blinded Randomised Controlled Trial (RCT) will be conducted, with follow-up at 6 and 9 months. Participants will be randomly assigned to either the control group (usual care) or intervention group (a 6-session, group-based SPACE for COPD© self-management programme delivered over 5 months). The primary outcome is change in COPD Assessment Test at 6-months.

A discussion session will be conducted with HCP's who deliver the intervention to discuss and gain insight into any potential facilitators/barriers to implementing the

For peer review only - http://bmjopen.bmj.com/site/about/guidelines.xhtml

intervention in practice. Furthermore, we will conduct semi-structured focus groups with intervention participants to understand feasibility and acceptability. All qualitative data will be analysed thematically.

Ethics and Dissemination

The project has received a favourable opinion from South Hampshire B Research Ethics Committee, REC reference: 14/SC/11/69 and full R&D approval from the University Hospitals of Leicester NHS Trust: 152408.

Study results will be disseminated through appropriate peer reviewed journals, national and international respiratory/physiotherapy conferences, via the Collaboration and Leadership in Applied Health Research and Care and through social media.

Trial registration: ISRCTN17942821.

Protocol Version: 11 18.11.2015

Key words: COPD, self-management support, primary care

For peer review only

Strengths and Limitations:

- The burden of COPD is significant to both the health service and the individual. Supported Self-management is important but options are limited for those with COPD. This study explores a group based supported self-management programme for individuals with COPD.

For peer review only - http://bmjopen.bmj.com/site/about/guidelines.xhtml

- This is a pragmatic trial where the study intervention (a group-based self-management support intervention for people with COPD) will be delivered and supported by healthcare professionals in community settings. The study has been designed to align with how the intervention might be delivered in routine clinical practice.
- Our follow-up period is 3 months post intervention. Unfortunately, due to funding constraints we are unable to carry out a longer-term follow-up.'

For peer review only

For peer review only - http://bmjopen.bmj.com/site/about/guidelines.xhtml

INTRODUCTION

COPD is the third leading cause of death worldwide, and is associated with considerable disability, impaired quality of life and high utilisation of healthcare resources (1). Symptoms and manifestations of the disease can be modified by adopting appropriate health behaviours, including but not limited to, exercise, physical activity, smoking cessation, anxiety management, breathing control, medication adherence and exacerbation management (2). Acknowledging the importance of the role of the patient in adopting these behaviours, there has been a shift in attitude from a traditional paternalistic model of care towards a more collaborative approach for chronic disease management. The NHS Five Year Forward View's aim is for the NHS to become better at helping people to manage their own health by staying healthy, making informed choices of treatment, managing conditions and avoiding complications (3). Inevitably, the patient is predominantly responsible for administering their own care, and making choices about health behaviours which will affect their outcomes. Self-management support aims to inform and support patients in making these choices. National and international guidelines for the management of COPD suggest that self-management support should be provided for people with COPD, though at present evidence for how and when that support should be delivered is less robust (2).

Reports in the literature describe programmes that have targeted interventions for patients who have been hospitalised with a COPD-related admission, often with the primary ambition of reducing future admissions (4). These studies have had little impact on readmission. Arguably, the offer of supported self-management should be offered earlier in the disease trajectory. Other COPD self-management programmes

For peer review only - http://bmjopen.bmj.com/site/about/guidelines.xhtml

beyond the UK have been described in a stable population. Although the models of care delivered are quite heterogeneous (5), with some programmes providing up to two years of weekly supervised exercise training and education (3, 6-8). The infrastructure and resources required to provide such comprehensive support means they are unlikely to be deliverable to the breadth of the COPD population in the UK. In order to address this, we previously developed and tested a new self-management programme which offered a 'light touch' approach so that it could be provided on a larger scale.

A Self-management Programme of Activity Coping and Education – SPACE FOR COPD© – aims to support people with COPD in managing day-to-day tasks, minimise symptom burden, provoke health enhancing behaviour change and enhance emotional well-being. The programme is structured around the SPACE FOR COPD© manual, which combines both generic self-management skills and disease-specific tasks. Pilot testing assessed the feasibility and acceptability of the intervention to patients (9), and a fully powered randomised controlled trial assessed the efficacy of the intervention in primary care (10), powered for change in symptom burden measured by the self-reported Chronic Respiratory Questionnaire (CRQ-SR) dyspnoea domain at six months. In these studies the SPACE FOR COPD© manual was introduced to patients during an initial consultation with a healthcare professional (using motivational interviewing techniques), followed by two telephone calls during the subsequent six weeks. Secondary outcomes included other domains of the CRQ-SR, shuttle walking tests, disease knowledge, anxiety, depression, self-efficacy, smoking status and healthcare utilisation measured at baseline, six weeks and six months follow-up. Results demonstrated significant short-term improvements

For peer review only - http://bmjopen.bmj.com/site/about/guidelines.xhtml

in CRQ-SR dyspnoea, anxiety, fatigue and emotion scores, exercise performance, and disease knowledge. At six months, anxiety, exercise performance and smoking status outcomes remained significantly different between the intervention group and the usual care group, though there was no between-group difference in change in CRQ-SR dyspnoea.

Implementation focussed work carried out following these studies with healthcare professionals in primary care and local Clinical Commissioning Groups (CCGs), demonstrated considerable interest in delivering the SPACE FOR COPD© intervention as a group-based intervention rather than on a one-to-one basis. The most common theoretical rationale underpinning delivery of group-based self-management support interventions is Social Cognitive Theory/Social Learning Theory (11, 12). Bandura's (1977, 1997) social learning theory posits that behaviour is influenced by beliefs about one's ability to perform a particular behaviour (self-efficacy expectations), beliefs about the effectiveness of the behaviour (e.g., the advantages and disadvantages of performing this behaviour; outcome expectations) and learning through social observation (including social norms, social support or pressure, and the behaviours of others). Peer support and use of other patients as role models are approaches grounded in this theory, and directly applicable to group-based self-management support interventions.

Delivery of SPACE for COPD© as a group-based intervention allows for several face-to-face contacts between patients and healthcare professionals over a number of sessions. These contacts could be spread out further across a longer period, which may be more successful in maintaining behaviour change. Furthermore,

For peer review only - http://bmjopen.bmj.com/site/about/guidelines.xhtml

having earlier sessions closer together in time allows group cohesion to take place, an important factor in optimising group dynamics (13).

The SPACE for COPD© intervention has also previously been delivered by a member of the research team rather than by existing clinical services. If the group-based intervention were to be implemented in primary care following the current study, importance would be placed on delivery by healthcare professionals in a format that is feasible and acceptable to healthcare professionals, health service providers and to patients. Understanding how this intervention can be delivered within existing health services and identifying key barriers and facilitators to its implementation is an important next step in the development of this complex intervention.

Aims and objectives of the study

1. To examine whether group-based delivery of SPACE FOR COPD©, with sustained support, improves patient outcomes following the intervention compared to a control group.
2. To explore feasibility, acceptability and efficacy of the intervention to be delivered and supported by HCP's. This will be done by:
    a. Exploring HCP's experiences of delivering the intervention and identify any barriers to delivery in practice.
    b. Understanding, from the patient perspective, the feasibility and acceptability of the SPACE for COPD© intervention delivered by HCPs in a group-based, community setting.

For peer review only - http://bmjopen.bmj.com/site/about/guidelines.xhtml

METHODS/DESIGN

Study design

The trial is a prospective, multi-site, single (assessor) blinded randomised controlled trial comparing a community based, HCP led, group-based self-management programme based on the SPACE for COPD© manual with usual care. The design of the study and flow of participants is described in figure 1. The study will be run across Leicestershire and Rutland, and a total of 150 participants will be recruited (75 in the intervention group and 75 in the control group).

Recruitment of participants

We will recruit participants with COPD, who will be identified from primary care (GP) COPD registers and from patients who respond to a poster advertisement that will be displayed at GP practices and hospitals. We will also recruit participants from the following areas within the Respiratory Biomedical Research Unit at University Hospitals of Leicester:

- Those who have been involved in previous research trials who have agreed to be contacted again; or
- Those who were unsuitable for previous research trials, but who agreed to be contacted about future research trials for which they might be eligible.

Participant invitation

Eligible individuals identified as having an established diagnosis of COPD are sent an invitation letter, a patient information sheet about the study, and a reply slip. For those recruited directly from primary care, the invitation letters are sent by the primary care practice where the search was conducted. For those recruited from

For peer review only - http://bmjopen.bmj.com/site/about/guidelines.xhtml

existing databases, the invitation is sent from the Principal Investigator of the study. Individuals who are interested in taking part are asked to return a reply slip directly to the SPACE for COPD© research team. Interested participants are then contacted via their preferred contact method and an appointment is arranged for a baseline visit.

Eligibility criteria

Participants are eligible for the trial if they have:

- An established diagnosis of Chronic Obstructive Pulmonary Disease (COPD) as defined by The Global Initiative for Chronic Obstructive Lung Disease (GOLD) criteria.

Patients are excluded from participating in the trial if they are:

- Unable to participate in the exercise component of the SPACE for COPD© programme due to neurological, locomotive, or psychiatric disability
- Unable to participate in the exercise component of the SPACE for COPD© programme due to other comorbidities where exercise would be a contraindication (for example, unstable angina).
- Unable to read/write English to the level of an eight year old
- Unwilling to be randomised
- Previous participants of Pulmonary Rehabilitation or have received the SPACE for COPD© manual in the previous 12 months.

Randomisation

Once participants have consented to take part in the study and spirometry has confirmed a COPD diagnosis, participants are randomised by an un-blinded member

For peer review only - http://bmjopen.bmj.com/site/about/guidelines.xhtml

of the study team using an online randomisation tool (sealed envelope;(14). Individuals are randomised (1:1) to the control group or the intervention (SPACE FOR COPD© group-based self-management programme) group. The system randomises patients in random permuted blocks. This allows for the 1:1 ratio, but due to the random permuted blocks of 2, 4, or 6 ensures full randomisation. Simple randomisation has been chosen as there has been no requirement to stratify by age, gender, location, or other variables. Participants are immediately informed of their allocated treatment by an un-blinded member of the study team. Un-blinding is permissible in the case of medical emergencies (e.g., cardiac arrest) or patients being admitted to hospital for an exacerbation.

Study Interventions

Usual Care (Control Group)

Participants in the control group will continue with any usual check-ups/reviews and there will be no additional care provided or removed from their current access. If patients are referred to pulmonary rehabilitation in the duration of their time in the study, they will not be denied access to the programme; however, they will not be included in the final analysis due to the use of ‘intention-to-treat’ analysis. No additional advice, information or recommendations will be provided to participants in this group.

For peer review only - http://bmjopen.bmj.com/site/about/guidelines.xhtml

SPACE for COPD© group-based self-management programme

Participants in the intervention group receive a SPACE for COPD© manual and asked to attend the SPACE for COPD© group-based self-management programme usually within one month of their baseline appointment. The aim of the SPACE for COPD© programme is to support people with COPD in managing day-to-day tasks, minimise symptom burden, provoke health enhancing behaviour change and enhance emotional well-being. The programme is structured around the SPACE FOR COPD© manual, which combines both generic self-management skills and disease-specific tasks. The programme is facilitated by two trained healthcare professionals (e.g., physiotherapists, respiratory specialist nurses, occupational therapists, health psychologists) to groups of up to 10 participants, and delivered through six, two hour sessions, over a five month period. These sessions will be held at community venues, at times and locations to suit participants of the group to increase retention. Participants are liaised with in regards to preferences on timings and location of the group sessions to increase retention, and engagement in the intervention. The content of the programme and accompanying self-management components (15), are presented in Tables 1 and 2. Participants are provided with a contact number for at least one of the facilitators throughout the course of the programme in case they have any further queries/are unable to attend any sessions.

Participants will also be asked to complete the exercise component of the manual at home in their own time. A full description of the rationale, development and efficacy of the work underpinning the SPACE for COPD© manual is detailed elsewhere (9). The intervention will be offered over a period of 2 years (the duration of the active

For peer review only - http://bmjopen.bmj.com/site/about/guidelines.xhtml

component of the intervention within the study) and will only be offered as part of the research, not routine practice within the community.

For peer review only

For peer review only - http://bmjopen.bmj.com/site/about/guidelines.xhtml

Table 1: SPACE for COPD© self-management programme session outline

**Session 1 (Week 1)**	**Introduction to SPACE FOR COPD©**
1	Welcome and introductions
2	Group responsibilities
3	What does it mean to have COPD?
4	What is self-management?
5	How to use the SPACE for COPD manual
6	Goal setting
7	Home activities for next session
8	Summary and close
**Session 2 (Week 2)**	**Introducing exercise and managing shortness of breath**
1	Welcome back
2	Solution focused goal feedback
3	Managing shortness of breath
4	Introduction to the walking programme
5	Goal setting
6	Activities for next session
7	Summary and close
**Session 3 (Week 4)**	**Continuing Exercise and Saving Energy**
1	Welcome back
2	Solution focused goal feedback
3	Saving your energy
4	Strength training
5	Goal setting
6	Home activities for next session
7	Group discussion
8	Summary and close
**Session 4 (Week 8)**	**Managing Stress and Emotions and the COPD Action Plan**
1	Welcome back
2	Solution focused goal feedback
3	Managing stress and emotions
4	Action plans
5	Goal setting
6	Activities for next session
7	Summary and close
**Session 5 (Week 14)**	**Question and Answer**
1	Welcome back
2	Solution focused goal feedback
3	Question and Answer
4	Goal setting
5	Activities for next session
6	Summary and close
**Session 6 (Week 20)**	**Keeping going from here**

For peer review only - http://bmjopen.bmj.com/site/about/guidelines.xhtml

1	Welcome back
2	Solution focused goal feedback
3	Hobbies
4	Maintaining exercise
5	Sharing success
6	Summary and close

For peer review only

For peer review only - http://bmjopen.bmj.com/site/about/guidelines.xhtml

Table 2: Taxonomy components present in the SPACE for COPD© facilitator manual, elaboration of the techniques under each component, direct examples from the SPACE for COPD© facilitator manual and the dose of the component

**Taxonomy component**	**Elaboration**	**Examples from the SPACE for COPD© facilitator manual (*dose*)**
**A2. Information about available resources**		Participants are provided with information throughout the programme (*every week*).
**A3. Provision of/agreement on specific clinical action plans and/or rescue medication**		*Session 4:* Action plans (*session 4 only*).
**A6. Practical support with adherence (medication or behavioural)**	Walking and strength training diaries are provided for participants and discussed during sessions.	Walking and strength training diaries are provided for participants and discussed during solution focused goal feedback at the beginning of sessions 3-6 (*sessions 3-6*).
**A8. Safety netting**	Participants are able to call programme facilitators between sessions if needed	Participants are provided with contact details for programme facilitators who they can call if needed (*this is a constant throughout the programme*).
**A11. Training/rehearsal for practical self-management activities**	Including:<br>• managing shortness of breath<br>• saving your energy	*Session 2:* Managing shortness of breath (*session 2 only*).<br>*Session 3:* Saving your energy (*session 3 only*).
**A12. Training/rehearsal in psychological strategies**	Including:<br>• goal setting (including action planning)<br>• solution focussed goal feedback<br>• problem solving<br>• self-reward and social reward<br>• managing stress and emotions	Goal setting activity (including action planning) and solution focussed goal feedback (*once every week*).<br><br>Problem solving (*this is a constant throughout the programme*).<br><br>*Session 4:* Managing stress and emotions (*session 4 only*).
**A13. Social support**	Including:<br>• practical support<br>• emotional support	Participants are encouraged to share experiences, advice, ideas and support each other (*this is a constant throughout the programme*).

For peer review only - http://bmjopen.bmj.com/site/about/guidelines.xhtml

**A14. Lifestyle advice and support**	Including:<br>• introduction to the walking programme<br>• strength training<br>• hobbies<br>• maintaining exercise	*Session 2:* Introduction to the walking programme (*session 2 only*).<br><br>*Session 3:* Strength training (*session 3 only*).<br>*Session 6:* Hobbies (*session 6 only*).<br>*Session 6:* Maintaining exercise (*session 6 only*).

For peer review only

For peer review only - http://bmjopen.bmj.com/site/about/guidelines.xhtml

Intervention fidelity

Intervention facilitators are registered health professionals (physiotherapists, respiratory nurse specialists, health psychologists). In total, 8 registered health professionals will be trained to deliver the SPACE for COPD© group-based intervention by two Health Psychologists.

All facilitators attended a one day training course to ensure that they understood the theories and philosophy underpinning the SPACE for COPD© group-based programme and the content and resources used within it. All facilitators were given a facilitator manual to support their delivery of the programme and given the opportunity to practise delivering at least one activity from the manual during the training session.

Quality Assurance

Quality assurance will be undertaken to assess delivery of intervention content and educational style. Intervention fidelity checklists for intervention facilitators and trained observers have been specifically designed for the study. Intervention facilitators will complete checklists at the end of each self-management group session, and one of the trainers will observe one session per self-management group, completing their own checklist. Intervention facilitators will receive written and verbal feedback from the trained assessor.

For peer review only - http://bmjopen.bmj.com/site/about/guidelines.xhtml

Study Outcomes

Data will be collected during baseline, 6-month and 9-month appointments at the Leicester Respiratory Biomedical Research Unit by trained members of the study team. Data are collected following standardised operating procedures. Written informed consent is obtained from all participants prior to the commencement of data collection. Details of all clinical assessments and outcome measures are provided in table 3. General practitioners are informed of patients' participation in the trial and any relevant results. Any serious adverse events will be reported to the sponsor and patients' ability to exercise safely will be monitored.

For peer review only

For peer review only - http://bmjopen.bmj.com/site/about/guidelines.xhtml

Table 3: Details of study clinical assessments and outcome measures at all appointments

Baseline appointment (blinded and un-blinded study team members)	6-month appointment (blinded study team member)	9-month appointment (blinded or un-blinded study team member)
Consent<br>Collection of demographic details and medical history<br>Blood Pressure<br>Spirometry<br>Randomisation*<br>Questionnaires (CAT, EQ-5D, CRQ-SR, BCKQ, PAM, HADS)*<br>Shuttle walking tests* (intervention participants only): 2xISWT; 1xESWT<br>Participants given Senswear activity monitor to wear for 7 days*	Check consent<br>Questionnaires (CAT, EQ-5D, CRQ-SR, BCKQ, PAM, HADS)<br>Shuttle walking tests†<br>Participants given Senswear activity monitor to wear for 7 days	Check consent<br>Questionnaires (CAT, EQ-5D, CRQ-SR, BCKQ, PAM, HADS)<br>Shuttle walking tests<br>Participants given Senswear activity monitor to wear for 7 days

Key:
CAT: COPD Assessment Test; EQ-5D: European Quality of Life-5 Dimensions; CRQ-SR: Chronic Respiratory Questionnaire; BCKQ: Bristol COPD Knowledge Questionnaire; PAM: Patient Activation Measure; HADS: Hospital Anxiety and Depression Questionnaire.
ISWT: Incremental Shuttle Walking Test; ESWT: Endurance Shuttle Walking Test
*Carried out by an un-blinded member of the study team
†ESWT carried out by an un-blinded member of the study team

For peer review only

Primary Outcome

The primary outcome is health status, as measured by the COPD Assessment Test (CAT;(16) at 6-months post baseline. This measure was chosen due to ease of use in clinical practice compared to the Chronic Respiratory Questionnaire (as used in the previous RCT). The CAT is a validated, short (8-item) and simple patient completed questionnaire, and assesses globally the impact of COPD (cough, sputum, dyspnea, chest tightness) on health status. The CAT is scored 0-5 with a range of 0-40; scores of 0-10, 11-20, 21-30, 31-40 represent mild, moderate, severe or very severe clinical impact.

Secondary Outcomes

Clinical measures

Exercise Capacity

Maximal exercise capacity will be measured with the incremental shuttle walk test (ISWT) according to the protocol of Singh et al.,(17) using a 10-m course. According to current standard, an individual change of at least 47.5 m is considered clinically important (18). Endurance capacity will be measured with the endurance shuttle walk test (ESWT) using a 10-m course and a walking speed of 85% of the maximal ISWT walking speed (19).

Physical Activity

Physical activity is assessed using physical activity monitors. The ‘Bodymedia Sensewear’ (APC Cardiovascular, UK) activity monitor is a biaxial accelerometer that

For peer review only - http://bmjopen.bmj.com/site/about/guidelines.xhtml

can report a number of parameters including step count and energy expenditure. We will also use this data to assess compliance to the physical activity recommendation of undertaking at least 150 minutes of moderate intensity physical activity per week in bouts of at least 10 minutes. Participants are asked to wear the activity monitor on the back of their right arm for seven consecutive days (24 hours a day if possible) following their baseline, 6-month and 9-month visits.

Questionnaires

Health related quality of life

Health related quality of life data will be measured using the European Quality of Life-5 Dimensions (EQ-5D;(20,21)). The EQ-5D is a standardized questionnaire that was developed for use as a measure of health outcomes and defines health in terms of five dimensions: mobility, self-care, usual activities, pain or discomfort, and anxiety or depression.

Chronic Respiratory Questionnaire (CRQ-SR)

Disease specific health related quality of life (HRQoL) will be measured by the self-administered standardised CRQ-SR (22). An individual change of at least 0.5/domain (dyspnoea, fatigue, emotional functioning, mastery) is considered clinically important (23). There is both an initial and follow-up version depending on time of administration.

For peer review only - http://bmjopen.bmj.com/site/about/guidelines.xhtml

Anxiety and Depression

Depression and anxiety will be measured using the Hospital Anxiety and Depression (HADS) Scale, to produce independent subscales for anxiety and depression (24). The HADS is a self-report rating scale of 14 items on a four-point Likert scale range (0–3). The HADS is a validated and a widely used questionnaire for screening for the separate dimensions of anxiety and depression and possible occurrence of anxiety and depression from patients (25) and the general population (26). It measures anxiety and depression (seven items for each subscale). Cronbach's coefficient was 0.884, which indicates good reliability. Published cut-off scores for clinically relevant indications of depression and anxiety recommend a score of 8 for each subscale (27).

Patient Activation

Patient activation (participants' knowledge, skill, and confidence for managing their own health and health care) will be measured using the Patient Activation Measure (PAM;(28). This is a 13-item patient-reported measure that has been validated in the United Kingdom as powerful and reliable measure of patient activation. Participants indicate their level of agreement on a four-point scale (strongly disagree to strongly agree) and responses are added to yield a raw score between 13 and 52. The raw score is calibrated to an activation score between 0 and 100 (the higher the score the higher the level of activation), which is then used to classify participants into one of four levels of activation (level 1: low activation; level 4: high activation).

For peer review only

COPD Knowledge

The Bristol COPD Knowledge Questionnaire (BCKQ) will be utilised to understand patients' informational requirements and understanding and their knowledge base of COPD (29). The questionnaire is comprehensive and goes into detail, regarding various aspects of COPD, for example epidemiology, signs and symptoms, and exacerbations and treatment.

Outcomes to assess feasibility and acceptability of trial parameters

We will use the following outcomes to assess the feasibility and acceptability of trial parameters:

Screening

Defined as the number of packs sent out to patients from GP practices and assessed for eligibility using inclusion/exclusion criteria by a study researcher.

Eligibility

Calculated by dividing number of people screened for eligibility by those who meet the inclusion criteria.

Consent

Defined as the proportion of people with COPD who met inclusion criteria, and were therefore eligible, who went on to consent in writing to participate in the study.

For peer review only - http://bmjopen.bmj.com/site/about/guidelines.xhtml

Group characteristics

Group characteristics (e.g., age, gender, GOLD stage, MRC, exercise capacity, physical activity) will be compared between completers and non-completers.

Retention

Defined as the number of participants who remain in the study and do not drop-out.

Study Completion

Defined by the number of participants who complete the COPD Assessment Test. Completion rates will be calculated at baseline, 6-month and 9-month follow-up.

Intervention adherence and completion rates

This will be measured by summing the total number of self-management programme sessions attended by participants allocated to the intervention group. We will also look at the average group size across each of the six self-management programme sessions and compare with the number of participants allocated to each of the self-management programme groups.

Qualitative Data Collection

Those participants allocated to the intervention group are invited to take part in qualitative focus groups at the end of the SPACE for COPD© intervention. Focus groups have been chosen due to their generation of information on collective views, and the meanings that lie behind those views. The aim of the focus groups will be to understand participants' experiences of the group-based self-management programme. More specifically, the data we collect will inform:

For peer review only - http://bmjopen.bmj.com/site/about/guidelines.xhtml

- Acceptability and usefulness of the programme to participants in this format and over this time period;
- The content of the intervention;
- Approaches to recruitment.

Focus groups will be conducted with each self-management programme group, with between 3 and 10 participants (number dependent on each group size). This difference in participant numbers allows for participant opinions to be gathered even if a small group is encountered (e.g., due to drop-out). Although three is a very small number for a focus group, it allows all participant opinions to be gathered, regardless of group size. Participants will be familiar with one another (which can help facilitate discussion or the ability to challenge each other comfortably) as they have attended multiple group sessions together. Purposive sampling will be employed to recruit intervention participants. Audio-recorded focus group discussions (approximately 60 minutes) will be conducted face-to-face between each participant group, an experienced interviewer and an observer/note-taker. Focus groups will be carried out at the end of the last group session for participant ease, as discussed with study patient representatives. We will prompt participants allocated to each self-management group of the focus group discussion prior to the last session in the attempt to gain experiences from as many participants as possible, regardless of the number of sessions attended in total. Focus groups will be transcribed verbatim by a professional transcriber, with identifiable information removed. Focus group questions have been devised based upon relevant literature and experience of the team.

For peer review only

Healthcare professionals delivering the SPACE for COPD© self-management support intervention will be invited to participate in a meeting to discuss aspects of feasibility and acceptability, such as gaining insight into any potential facilitators/barriers to implementing the intervention in practice (and derive practical recommendations for doing so). Minutes will be taken during the discussion and anonymised.

For peer review only

Data Analysis

Study Power

The power calculation was based on the primary outcome at 6-months (30, 31). To detect a mean ±SD between-group difference of 2.5±5.0 in the change in CAT with 80% power, 60 people per group are required (α=0.05, two-tailed). In anticipation of a possible 25% attrition rate, the total sample size was increased to 75 per group (150 in total).

Quantitative Analysis

This will primarily be completed on an intention-to-treat analysis. All quantitative data will be assessed for normality and analysed using appropriate parametric and non-parametric statistics (e.g., within and between measures t-tests and ANOVAs), statistical significance will be set at p=0.05. Secondary per protocol analyses will be carried out.

For peer review only - http://bmjopen.bmj.com/site/about/guidelines.xhtml

A post hoc analysis will be carried out, which will exclude patients in either arm that received pulmonary rehabilitation as part of their usual care. We would anticipate that patients who participate within the study, will not require Pulmonary Rehabilitation. However, due to Pulmonary Rehabilitation being a part of 'best' usual care, this will not be withheld from the patient.

Quantitative data for all outcomes will be transcribed from the CRF onto an electronic database. A statistical software package will be used to carry out quantitative analyses. Predictive Analaytics Software (SPSS) will be used to analyse the data, the license for which is provided by University Hospitals of Leicester NHS Trust. Continuous variables will be presented as mean and standard deviation (SD) or median and inter quartile range (IQR), and categorical data will be presented as frequencies and percentages. Data will be checked for normality and appropriate parametric and nonparametric tests will be used. Any baseline differences will be adjusted for. Any missing data will be imputed, and both intention-to-treat and per protocol analyses will be conducted.

We have not secured funding for a healthcare utilisation analysis but would anticipate further CLAHRC (Collaboration and Leadership in Applied Health Research and Care) funding if the trial is clinically effective.

For peer review only - http://bmjopen.bmj.com/site/about/guidelines.xhtml

Qualitative Analysis

The focus groups will be analysed using Thematic Analysis (32) supported by Nvivo software (version 9). This approach follows six distinct stages: familiarization with data; generating initial codes; searching for themes; reviewing themes; defining and naming themes and producing the report. The psychologist and the physiotherapist will carry out initial coding and a sample of interviews will be coded by another member of the team to ensure consistency and to enhance interpretive authenticity. Throughout the data analysis, the team will meet to discuss and review emerging themes and search for accounts that provide contesting views of the same phenomena or identify different phenomena. Our patient representatives will be invited to comment on our (anonymised) findings throughout the analysis process to ensure interpretations made by researchers stay close to the direct experience of patients (33).

All patient information that is collected during the course of the research will be kept strictly confidential. Any information about the patient which leaves the hospital will have their name and address removed. Participants will not be identified in any subsequent written material. Results will be reported in such a way that completely preserves confidentiality.

For peer review only - http://bmjopen.bmj.com/site/about/guidelines.xhtml

ETHICS AND DISSEMINATION

Ethics

The trial is sponsored by the University Hospitals of Leicester NHS Trust (study number 152408) and ethical approval was granted by the Hampshire B Research Ethics Committee (REC reference: 14/SC/11/69). Protocol amendments will be approved by the ethics committee and regulatory authorities as per current guidelines and will be communicated to relevant parties by the study team.

Dissemination

We plan to publish the results of the study in peer-reviewed journals and present them at appropriate national and international respiratory and physiotherapy conferences. Social media will be utilised to disseminate information and summaries of results to a wider population.

The CLAHRC East Midlands, is a large organisation which strives to improve health outcomes in the population across the East Midlands through delivering high quality, world class research. This organisation will be used to further disseminate results within the East Midlands. We also hope to provide a summary of results to the study participants. We also plan to hold a participant dissemination day towards the end of the study. This will enable participants to contribute to the final report and other result dissemination activities.

The institution also has an active and dynamic Public Involvement group for pulmonary and cardiovascular rehabilitation. The group will be used to create and co-ordinate strategies for further disseminating the results into the public domain.

For peer review only - http://bmjopen.bmj.com/site/about/guidelines.xhtml

The study may also be subject to internal and further external audits to ensure safety of the trial.

CONCLUSION

The importance of self-management is widely acknowledged and opportunities should be maximised from the time of diagnosis through to more severe disease. Opportunities to improve self-management skills should be embedded in a pulmonary rehabilitation programme. In the future, there may be an opportunity to explore the value of the SPACE for COPD© programme alongside rehabilitation, or indeed, an alternative for those unwilling or unable to attend. However, for those with milder disease there is no provision for a structured supported self-management programme in the UK. Evidence suggests that the SPACE for COPD© package is effective when delivered on an individual basis (10). This study examines its effectiveness as a group-based intervention in the community, as an alternative supported self-management strategy, which importantly allows patient choice.

FUNDING

The research is funded by the National Institute for Health Research (NIHR) Collaboration for Leadership in Applied Health Research and Care East Midlands (CLAHRC EM), and took place at the University Hospitals of Leicester NHS Trust. Support is also provided by the NIHR Leicester Respiratory Biomedical Research Unit (BRU). The views expressed are those of the authors and not necessarily those of the NHS, the NIHR or the Department of Health.

For peer review only - http://bmjopen.bmj.com/site/about/guidelines.xhtml

Roles of the funder: Management of staff, study progress, reporting and dissemination activities.

Study Sponsor

Carolyn Maloney. University Hospitals of Leicester NHS Trust, 0116 258 4109

Steering Committee and Acknowledgements

East Leicestershire CCG - Sue Price; Leicestershire Partnership Trust: Karen Moore, Alex Woodward, Gillian Doe; West Leicestershire CCG: Jake Cooke; Public Involvement: Patricia Overty, Freda Smart

Study Timeline

Study start 02/2015, recruitment 20 months, study end 06/2017

AUTHORS CONTRIBUTIONS

SS is the principal investigator of the SPACE for COPD© study. KM, SS, SB, LA, LHW, SH, JB and CB were involved in the development of the intervention and design of the trial. CB, SS, PK and THD have been involved in drafting the work or revising it critically for important intellectual content and have given the final approval of the version published.

COMPETING INTERESTS

None

Data sharing statement: Additional unpublished data from the study is still being collected and analysed and is only available to members of the study team.

For peer review only

REFERENCES

(1) World Health Organisation. Chronic Obstructive Pulmonary Disease (COPD). 2016; Available at: http://www.who.int/respiratory/copd/en/. Accessed 05/06/2016.

(2) National Institute for Health and Care Excellence. Chronic obstructive pulmonary disease in over 16s: diagnosis and management. 2010;CG101.

(3) NHS England. Five year forward view. October 2014. (report) https://www.england.nhs.uk/wp-content/uploads/2014/10/5yfv-web.pdf

(4) Purdy S. Avoiding hospital admissions: What does the research evidence say? 2010. The King's Fund (report). https://www.kingsfund.org.uk/sites/files/kf/Avoiding-Hospital-Admissions-Sarah-Purdy-December2010.pdf

(5) Zwerink M, Brusse-Keizer M, van der Valk PD, et al. Self management for patients with chronic obstructive pulmonary disease. Cochrane Database of Systematic Reviews. 2014.

(6) Effing T, Zielhuis G, Kerstjens H, et al. Community based physiotherapeutic exercise in COPD self-management: A randomised controlled trial. Respir Med 2011 3;105(3):418-426.

(7) Monninkhof E, van der Valk P, van der Palen J, et al. Effects of a comprehensive self-management programme in patients with chronic obstructive pulmonary disease. European Respiratory Journal 2003 European Respiratory Society;22(5):815-820.

For peer review only - http://bmjopen.bmj.com/site/about/guidelines.xhtml

(8) Bourbeau J, Julien M, Maltais F,et al. Reduction of hospital utilization in patients with chronic obstructive pulmonary disease: A disease-specific self-management intervention. Archives of Internal Medicine 2003 March 10;163(5):585-591.

(9) Apps LD, Mitchell KE, Harrison SL, et al. The development and pilot testing of the Self-management Programme of Activity, Coping and Education for Chronic Obstructive Pulmonary Disease (SPACE for COPD). International Journal of Chronic Obstructive Pulmonary Disease 2013;8:317-327.

(10) Mitchell KE, Johnson-Warrington V, Apps LD, et al. A self-management programme for COPD: a randomised controlled trial. European Respiratory Journal 2014 European Respiratory Society;44(6):1538-1547.

(11) Bandura A. Social Learning Theory. Englewood Cliffs, NJ: Prentice Hall; 1977.

(12) Bandura A. Self-efficacy: The exercise of control. New York: W.H. Freeman; 1997.

(13) Yalom I. Theory and practice of group psychotherapy. Fifth ed. New York: Basic Books; 2005.

(14) Sealed Envelope Ltd. Randomisation and and online databases for clinical trials. 2016; Available at: http://www.sealedenvelope.com/. Accessed 01/06, 2016.

(15) Pearce G, Parke HL, Pinnock H, et al. The PRISMS taxonomy of self-management support: derivation of a novel taxonomy and initial testing of its utility. Journal of Health Services Research & Policy 2016 April 01;21(2):73-82.

For peer review only - http://bmjopen.bmj.com/site/about/guidelines.xhtml

(16) Jones PW, Harding G, Berry P, et al. Development and first validation of the COPD Assessment Test. European Respiratory Journal 2009 European Respiratory Society;34(3):648-654.

(17) Singh SJ, Morgan M, Scott S, et al. Development of a shuttle walking test of disability in patients with chronic airways obstruction. Thorax 1992;47(12):1019-1024.

(18) Singh SJ, Jones PW, Evans R, et al. Minimum clinically important improvement for the incremental shuttle walking test. Thorax 2008 September 01;63(9):775-777.

(19) Revill SM, Morgan MDL, Singh SJ, et al. The endurance shuttle walk: a new field test for the assessment of endurance capacity in chronic obstructive pulmonary disease. Thorax 1999 March 01;54(3):213-222.

(20) EuroQol Group. EuroQol - a new facility for the measurement of health-related quality of life. Health Policy 1990;16(3):199-208.

(21) Rutten-van-Molken MP, Oostenbrink JB, Tashkin DP, et al. Does Quality of Life of COPD Patients as Measured by the Generic EuroQol Five-Dimension Questionnaire Differentiate Between COPD Severity Stages? Chest 2006 October;130(4):1117-1128.

(22) Williams JEA, Singh SJ, Sewell L, et al. Development of a self-reported Chronic Respiratory Questionnaire (CRQ-SR). Thorax 2001 December 01;56(12):954-959.

(23) Juniper EF, Guyatt GH, Willan A, et al. Determining a minimal important change in a disease-specific quality of life questionnaire. J Clin Epidemiol 1994 January 1994;47(1):81-87.

For peer review only

For peer review only - http://bmjopen.bmj.com/site/about/guidelines.xhtml

(24) Zigmond AS, Snaith RP. The Hospital Anxiety and Depression Scale. Acta Psychiatr Scand 1983;67(6):361-370.

(25) Al-Gamal E, Yorke J. Perceived breathlessness and psychological distress among patients with chronic obstructive pulmonary disease and their spouses. Nurs Health Sci 2014;16(1):103-111.

(26) Bjelland I, Dahl A, Haug T, et al. The validity of the hospital anxiety and depression scale: An updated literature review. Journal of Psychosomatic Research 2002;52(2):69-77.

(27) Hung C, Liu C, Wang S, et al. The cut-off points of the Depression and Somatic Symptoms Scale and the Hospital Anxiety and Depression Scale in detecting non-full remission and a current major depressive episode. Int J Psychiatry Clin Pract 2012 03/01;16(1):33-40.

(28) Donald M, Ware RS, Ozolins IZ, et al. The role of patient activation in frequent attendance at primary care: A population-based study of people with chronic disease. Patient Educ Couns 2011 5;83(2):217-221.

(29) White R, Walker P, Roberts S, et al. Bristol COPD Knowledge Questionnaire (BCKQ): testing what we teach patients about COPD. Chronic Respiratory Disease 2006 July 01;3(3):123-131.

(30) Jones PW, Harding G, Wiklund I, et al. Tests of the responsiveness of the copd assessment test following acute exacerbation and pulmonary rehabilitation. Chest 2012 July 1;142(1):134-140.

For peer review only - http://bmjopen.bmj.com/site/about/guidelines.xhtml

(31) Kon SSC, Canavan JL, Jones SE, et al. Minimum clinically important difference for the COPD Assessment Test: a prospective analysis. The Lancet Respiratory Medicine 2014 3;2(3):195-203.

(32) Braun V, Clarke V. Using thematic analysis in psychology. Qualitative Research in Psychology 2006;3(2):77-101.

(33) Boote J, Baird W, Beecroft C. Public involvement at the design stage of primary health research: A narrative review of case examples. Health Policy 2010 4;95(1):10-23.

For peer review only

For peer review only - http://bmjopen.bmj.com/site/about/guidelines.xhtml

Figure Legend:

Figure 1: Participant flow through the study

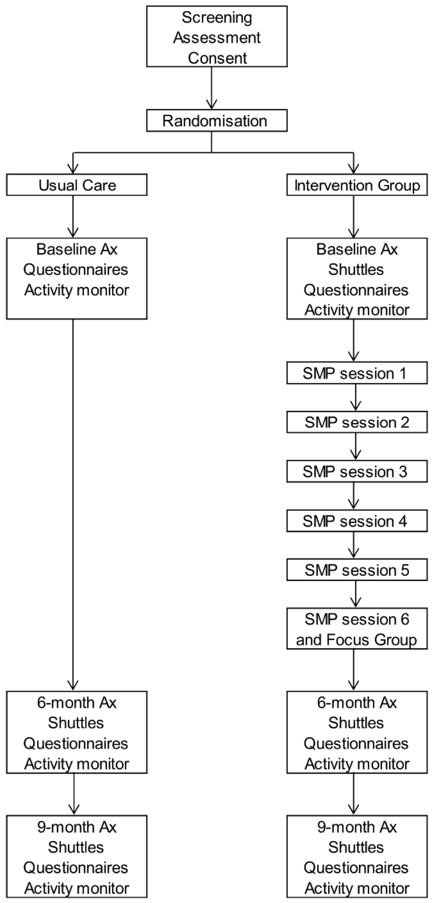

Figure 1: Participant flow through the study

210x297mm (300 x 300 DPI)

University Hospitals of Leicester NHS
NHS Trust

Glenfield Hospital
Groby Road
Leicester
LE3 9QP

Tel: 0116 287 1471
Fax: 0116 258 3950
Minicom: 0116 287 9852

CONSENT FORM for Patients
(Version 5 08/05/2015)

Identification Number for this study:

A self-management programme of activity coping and education - SPACE FOR COPD - in primary care: a pragmatic trial

Please initial box

1. I confirm that I have read and understand the information sheet version 5 (08/05/2015) for the above study and have had the opportunity to ask questions. ☐
2. I understand that my participation is voluntary and that I am free to withdraw at any time, without giving any reason, without my medical care or legal rights being affected. ☐
3. I understand that the research team will access my medical notes to obtain data that is relevant to this study, such as information about healthcare use and to follow up information about my health. I give the researchers' permission to access my medical notes for this purpose. ☐
4. I understand that my personal information may be stored, safely and securely, at University Hospitals of Leicester as well as at my GP practice. ☐
5. I agree for my GP to be informed of my participation and that results from some of the tests performed as part of this study may be sent to my GP. ☐
6. I understand that some of my data will be shared with Insignia Health and entered onto a secure database to help improve patient outcome measures, if it is anonymised. ☐
7. I agree to take part in the above study. ☐
8. I understand that all discussions at group sessions are confidential and I agree to respect this confidentiality. ☐
9. I agree to take part in a focus group discussion at the end of the self-management programme which will be recorded and transcribed for research purposes. I also agree that anonymised direct quotes can be used for research purposes**.** yes ☐ no ☐

Name of Patient	Date	Signature
Name of Person taking consent	Date	Signature

Consent form for patients Version 5 08/05/2015

For peer review only

University Hospitals of Leicester NHS
NHS Trust

Glenfield Hospital
Groby Road
Leicester
LE3 9QP

Tel: 0116 287 1471 ext 250 2762
or 250 2758
Fax: 0116 258 3950
Minicom: 0116 287 9852

A self-management programme of activity coping and education - SPACE FOR COPD - in primary care: a pragmatic trial (Version 5 08/05/2015)

You are being invited to take part in research study being conducted by University Hospitals of Leicester NHS Trust. Before you decide whether or not to take part it is important for you to understand why the research is being done and what it will involve. Please take time to read the following information carefully and discuss it with others if you wish. Ask us if there is anything that is not clear or if you would like more information. Take time to decide whether or not you wish to take part.

We have developed a self-management manual for patients with Chronic Obstructive Pulmonary Disease (COPD). This has been done with enormous help from patients like yourself. The manual covers issues such as drug and symptom management, exercise and nutrition at home.

In a previous study, we provided this manual for people to work through at home, with one initial meeting with a healthcare professional. While that study showed there were several benefits to having the manual, we would now like to see if the benefits will last longer if support from the healthcare professional is increased. Also patients have told us they would like the manual to be and supported in a peer group environment. This study, therefore, will test the effectiveness of using the manual within a group setting over a number of sessions.

We would like to know how effective the use of this manual may be when delivered and supported in a group environment. This will help us improve future patient care.

2. Why have I been invited?

As an individual with COPD you have been identified as a suitable participant by your GP or your healthcare professional. You might have also been involved in a previous research trial and agreed to be contacted again or agreed to be contacted about future trials for which you might be eligible. It is important we see how people progress using the manual we have developed compared to those who receive standard care from their GP or healthcare professional. This knowledge may help us develop and improve future services.

3. Do I have to take part?

It is up to you to decide whether or not to take part. If you do decide to take part you will be given this information sheet to keep and be asked to sign a consent form. If you decide to

For peer review only - http://bmjopen.bmj.com/site/about/guidelines.xhtml

take part you are still free to withdraw at any time and without giving a reason. Your decision to withdraw will not influence any medical care you receive.

4. If you agree to part what will happen?

Once you have provisionally agreed to take part in the research you will be contacted to arrange a date and time to discuss the project in more detail. You will be invited to attend an appointment which will take place at Glenfield Hospital. If you still wish to go ahead at this stage then we will ask you to sign a consent form to take part in the study. Following this we will do some lung function tests. These are tests where you blow out into a tube as hard as you can. This confirms that you do have a diagnosis of COPD. You will then be randomly allocated to one of two arms of the trial. Half of the people in the study will be randomly allocated to receive the self-management group programme. The other half will be randomly assigned to 'usual care' which means that we will not change any of your current treatment or care. You will be randomly allocated to one of these two arms of the study by a computer-based system. This means that we will not know before hand which arm of the study you will be in. It is important that this is done at random in order to keep the results of the study fair.

5. What do I have to do?

If you are assigned to the usual care group you will continue as normal with the treatment you receive from your GP and healthcare team. At your first visit with us, you will be requested to complete some questionnaires about your health status. These will take about 20 minutes to complete. We will also ask you to wear an activity monitor for the following 7 days at home. The monitor is a small device which is worn around your arm, and it measures your activity levels at home. We will arrange to collect this from you after you have finished wearing it. We will ask you to repeat the questionnaires and wearing of the activity monitor after 6 months, and then again 3 months later (9 months from starting the study and your final assessment). In addition to the questionnaires and activity monitor at 6 months and 9 months, we will ask you to attend the hospital to do some walking tests. These will be done at Glenfield Hospital. There will usually be three walking tests, which are done on a flat corridor, walking up and down a 10 metre course. It is usual to become breathless during these tests, however we will only ask you to do as much as you feel you can.

If you are assigned to the self-management group you will undergo the same assessments at the same time points as the usual care group. However, in addition to these we will also ask you to do the walking tests at all three time points (i.e. your first visit, 6 month visit and 9 month visit). These will all take place at Glenfield Hospital. In between your first visit and your 6 month visit we will ask you to attend the group sessions to introduce you to the self-management programme. These will take place at a community centre. There will be approximately between six and 10 people in each group, and all sessions will be led by 2 healthcare professionals. Each session will last approximately two hours. At your first session you will be given the SPACE FOR COPD manual, and each session thereafter will discuss a different topic about how to manage with COPD. You will be advised how you can increase your activities at home, improve management of your breathlessness and learn what to do if you develop a chest infection. You do not have to discuss anything within the group if you do not wish to, and everything discussed within the group will be kept confidential. At the end of the self-management programme we will also ask you to take part in a focus group discussion where you will have an opportunity to discuss your experiences of the programme and whether you thought it was useful to you. The focus group will last

For peer review only - http://bmjopen.bmj.com/site/about/guidelines.xhtml

approximately 1 hour. The discussions will be audio recorded and transcribed for research purposes.

During the study, and immediately afterwards the study team will access your medical notes to obtain long term follow up data (for example GP visits, hospital admissions).

6. Are there any risks, disadvantages or side-effects of taking part?

We do not expect there are any risks to taking part in this study.

One potential disadvantage to taking part is the time you give to the study. Your first visit may take up to two hours. Your six-month and nine-month visits may take up to one hour each.

Whilst completing the walking tests, you may become short of breath, however we expect this will recover once you sit and rest. Afterwards, you may experience some achiness in your muscles and feel tired. This is usually only lasts a day or two.

7. What are the possible benefits of taking part?

We hope that the research will aid you in your understanding of COPD and how you can improve your symptoms. Information gathered will inform both present and future research, aiming to provide better care for people living with COPD.

8. What if something goes wrong?

If you are harmed by taking part in this research project, there are no special compensation arrangements. If you are harmed due to someone’s negligence, then you may have grounds for a legal action but you may have to pay for it. Regardless of this, if you wish to complain, or have any concerns about any aspect of the way you have been approached or treated during the course of this study, the normal National Health Service complaints mechanisms would be available to you.

Should you wish to discuss any concerns about this study further, the Patient Information and Liaison Service is a point of contact for you. Their telephone number is 0808 178 8337.

9. Will my taking part in this study be kept confidential?

All information that is collected about you during the course of the research will be kept strictly confidential. Any information about you, which leaves the medical centre, will have your name and address removed so that you cannot be recognised from it. Some of your anonymised data will be shared with Insignia Health (a healthcare organisation) and entered onto a secure database to help improve patient outcome measures. This will be basic information, for example age, gender and some questionnaire data. Participants will not be identified in any subsequent written material. Results will be reported in such a way that completely preserves confidentiality. Data will be stored at Glenfield Hospital, and on University Hospitals of Leicester secure networks.

For peer review only - http://bmjopen.bmj.com/site/about/guidelines.xhtml

We will, with your consent inform your GP of your participation in the trial and share with your GP any relevant test results that have been made during your participation in the trial.

10. What will happen to the results of the research study?

The results of the study will be written in peer and lay journals, professional publications and presentations made at relevant conferences. Results will be reported in such a way that preserves confidentiality. All participants will also receive a summary of the results.

11. Who is organising and funding the research?

This study is being organised by staff employed at University Hospitals of Leicester NHS trust. It is being funded by a Collaboration and Leadership in Applied Health Research and Care East Midlands (CLAHRC).

12. Who has reviewed the study?

All research that involves the NHS is reviewed by an NHS Research Ethics Committee. This study has been reviewed and given a favourable opinion by Hampshire B research Ethics Committee. This does not guarantee that you will not come to any harm if you take part. However, approval means that the committee is satisfied that your rights will be respected, that any risks have been reduced to a minimum and balanced against possible benefits and that you have been given sufficient information of which to make an informed decision. The study has also been reviewed and is sponsored by University Hospitals of Leicester.

13. Contact for further information

If you have any concerns or other questions about this study or the way it has been carried out, you should contact the principal researcher Professor Sally Singh:

Contact for further information:

The research team
Centre for Exercise and Rehabilitation Science
Leicester Respiratory Biomedical Research Unit,
Glenfield Hospital,
Leicester,
LE3 9PQ

0116 2583035
sally.singh@uhl-tr.nhs.uk

Thank you for reading this
Yours faithfully

Sally Singh

For peer review only - http://bmjopen.bmj.com/site/about/guidelines.xhtml

SPIRIT

STANDARD PROTOCOL ITEMS: RECOMMENDATIONS FOR INTERVENTIONAL TRIALS

SPIRIT 2013 Checklist: Recommended items to address in a clinical trial protocol and related documents*

Section/item	Item No	Description	Addressed on page number
**Administrative information**			
Title	1	Descriptive title identifying the study design, population, interventions, and, if applicable, trial acronym	1
Trial registration	2a	Trial identifier and registry name. If not yet registered, name of intended registry	3
	2b	All items from the World Health Organization Trial Registration Data Set	N/A
Protocol version	3	Date and version identifier	3
Funding	4	Sources and types of financial, material, and other support	27 and 28
Roles and responsibilities	5a	Names, affiliations, and roles of protocol contributors	1
	5b	Name and contact information for the trial sponsor	28
	5c	Role of study sponsor and funders, if any, in study design; collection, management, analysis, and interpretation of data; writing of the report; and the decision to submit the report for publication, including whether they will have ultimate authority over any of these activities	28
	5d	Composition, roles, and responsibilities of the coordinating centre, steering committee, endpoint adjudication committee, data management team, and other individuals or groups overseeing the trial, if applicable (see Item 21a for data monitoring committee)	28

For peer review only - http://bmjopen.bmj.com/site/about/guidelines.xhtml

**Introduction**			
Background and rationale	6a	Description of research question and justification for undertaking the trial, including summary of relevant studies (published and unpublished) examining benefits and harms for each intervention	5-8
	6b	Explanation for choice of comparators	8
Objectives	7	Specific objectives or hypotheses	8
Trial design	8	Description of trial design including type of trial (eg, parallel group, crossover, factorial, single group), allocation ratio, and framework (eg, superiority, equivalence, noninferiority, exploratory)	9
**Methods: Participants, interventions, and outcomes**			
Study setting	9	Description of study settings (eg, community clinic, academic hospital) and list of countries where data will be collected. Reference to where list of study sites can be obtained	9
Eligibility criteria	10	Inclusion and exclusion criteria for participants. If applicable, eligibility criteria for study centres and individuals who will perform the interventions (eg, surgeons, psychotherapists)	10
Interventions	11a	Interventions for each group with sufficient detail to allow replication, including how and when they will be administered	11-16
	11b	Criteria for discontinuing or modifying allocated interventions for a given trial participant (eg, drug dose change in response to harms, participant request, or improving/worsening disease)	N/A
	11c	Strategies to improve adherence to intervention protocols, and any procedures for monitoring adherence (eg, drug tablet return, laboratory tests)	17
	11d	Relevant concomitant care and interventions that are permitted or prohibited during the trial	11
Outcomes	12	Primary, secondary, and other outcomes, including the specific measurement variable (eg, systolic blood pressure), analysis metric (eg, change from baseline, final value, time to event), method of aggregation (eg, median, proportion), and time point for each outcome. Explanation of the clinical relevance of chosen efficacy and harm outcomes is strongly recommended	18-24
Participant timeline	13	Time schedule of enrolment, interventions (including any run-ins and washouts), assessments, and visits for participants. A schematic diagram is highly recommended (see Figure)	19 & diagram 1

For peer review only - http://bmjopen.bmj.com/site/about/guidelines.xhtml

Sample size	14	Estimated number of participants needed to achieve study objectives and how it was determined, including clinical and statistical assumptions supporting any sample size calculations	24
Recruitment	15	Strategies for achieving adequate participant enrolment to reach target sample size	9-10
**Methods: Assignment of interventions (for controlled trials)**			
Allocation:			
Sequence generation	16a	Method of generating the allocation sequence (eg, computer-generated random numbers), and list of any factors for stratification. To reduce predictability of a random sequence, details of any planned restriction (eg, blocking) should be provided in a separate document that is unavailable to those who enrol participants or assign interventions	10-11
Allocation concealment mechanism	16b	Mechanism of implementing the allocation sequence (eg, central telephone; sequentially numbered, opaque, sealed envelopes), describing any steps to conceal the sequence until interventions are assigned	10
Implementation	16c	Who will generate the allocation sequence, who will enrol participants, and who will assign participants to interventions	10-11
Blinding (masking)	17a	Who will be blinded after assignment to interventions (eg, trial participants, care providers, outcome assessors, data analysts), and how	11 & 19
	17b	If blinded, circumstances under which unblinding is permissible, and procedure for revealing a participant's allocated intervention during the trial	11
**Methods: Data collection, management, and analysis**			
Data collection methods	18a	Plans for assessment and collection of outcome, baseline, and other trial data, including any related processes to promote data quality (eg, duplicate measurements, training of assessors) and a description of study instruments (eg, questionnaires, laboratory tests) along with their reliability and validity, if known. Reference to where data collection forms can be found, if not in the protocol	18-24
	18b	Plans to promote participant retention and complete follow-up, including list of any outcome data to be collected for participants who discontinue or deviate from intervention protocols	N/A

For peer review only - http://bmjopen.bmj.com/site/about/guidelines.xhtml

Data management	19	Plans for data entry, coding, security, and storage, including any related processes to promote data quality (eg, double data entry; range checks for data values). Reference to where details of data management procedures can be found, if not in the protocol	24-26
Statistical methods	20a	Statistical methods for analysing primary and secondary outcomes. Reference to where other details of the statistical analysis plan can be found, if not in the protocol	25
	20b	Methods for any additional analyses (eg, subgroup and adjusted analyses)	25
	20c	Definition of analysis population relating to protocol non-adherence (eg, as randomised analysis), and any statistical methods to handle missing data (eg, multiple imputation)	25
**Methods: Monitoring**			
Data monitoring	21a	Composition of data monitoring committee (DMC); summary of its role and reporting structure; statement of whether it is independent from the sponsor and competing interests; and reference to where further details about its charter can be found, if not in the protocol. Alternatively, an explanation of why a DMC is not needed	N/A
	21b	Description of any interim analyses and stopping guidelines, including who will have access to these interim results and make the final decision to terminate the trial	N/A
Harms	22	Plans for collecting, assessing, reporting, and managing solicited and spontaneously reported adverse events and other unintended effects of trial interventions or trial conduct	18
Auditing	23	Frequency and procedures for auditing trial conduct, if any, and whether the process will be independent from investigators and the sponsor	N/A
**Ethics and dissemination**			
Research ethics approval	24	Plans for seeking research ethics committee/institutional review board (REC/IRB) approval	26
Protocol amendments	25	Plans for communicating important protocol modifications (eg, changes to eligibility criteria, outcomes, analyses) to relevant parties (eg, investigators, REC/IRBs, trial participants, trial registries, journals, regulators)	26

For peer review only - http://bmjopen.bmj.com/site/about/guidelines.xhtml

Consent or assent	26a	Who will obtain informed consent or assent from potential trial participants or authorised surrogates, and how (see Item 32)	10 & 19
	26b	Additional consent provisions for collection and use of participant data and biological specimens in ancillary studies, if applicable	N/A
Confidentiality	27	How personal information about potential and enrolled participants will be collected, shared, and maintained in order to protect confidentiality before, during, and after the trial	26
Declaration of interests	28	Financial and other competing interests for principal investigators for the overall trial and each study site	28
Access to data	29	Statement of who will have access to the final trial dataset, and disclosure of contractual agreements that limit such access for investigators	29
Ancillary and post-trial care	30	Provisions, if any, for ancillary and post-trial care, and for compensation to those who suffer harm from trial participation	N/A
Dissemination policy	31a	Plans for investigators and sponsor to communicate trial results to participants, healthcare professionals, the public, and other relevant groups (eg, via publication, reporting in results databases, or other data sharing arrangements), including any publication restrictions	27
	31b	Authorship eligibility guidelines and any intended use of professional writers	N/A
	31c	Plans, if any, for granting public access to the full protocol, participant-level dataset, and statistical code	N/A
**Appendices**			
Informed consent materials	32	Model consent form and other related documentation given to participants and authorised surrogates	PIS and consent form attached separately
Biological specimens	33	Plans for collection, laboratory evaluation, and storage of biological specimens for genetic or molecular analysis in the current trial and for future use in ancillary studies, if applicable	N/A

*It is strongly recommended that this checklist be read in conjunction with the SPIRIT 2013 Explanation & Elaboration for important clarification on the items. Amendments to the protocol should be tracked and dated. The SPIRIT checklist is copyrighted by the SPIRIT Group under the Creative Commons "Attribution-NonCommercial-NoDerivs 3.0 Unported" license.

For peer review only - http://bmjopen.bmj.com/site/about/guidelines.xhtml
